# Cleavage fragments of the C-terminal tail of polycystin-1 are regulated by oxidative stress and induce mitochondrial dysfunction

**DOI:** 10.1016/j.jbc.2023.105158

**Published:** 2023-08-12

**Authors:** Hannah Pellegrini, Elizabeth H. Sharpe, Guangyi Liu, Eiko Nishiuchi, Nicholas Doerr, Kevin R. Kipp, Tiffany Chin, Margaret F. Schimmel, Thomas Weimbs

**Affiliations:** 1 Department of Molecular, Cellular, and Developmental Biology, University of California Santa Barbara, Santa Barbara, California, USA; 2 Department of Nephrology, Qilu Hospital, Cheeloo College of Medicine, Shandong University, Jinan, Shandong, China

**Keywords:** polycystic kidney disease, ADPKD, polycystin-1, PC1, mitochondria, metabolism, oxidative stress, ROS

## Abstract

Mutations in the gene encoding polycystin-1 (PC1) are the most common cause of autosomal dominant polycystic kidney disease (ADPKD). Cysts in ADPKD exhibit a Warburg-like metabolism characterized by dysfunctional mitochondria and aerobic glycolysis. PC1 is an integral membrane protein with a large extracellular domain, a short C-terminal cytoplasmic tail and shares structural and functional similarities with G protein–coupled receptors. Its exact function remains unclear. The C-terminal cytoplasmic tail of PC1 undergoes proteolytic cleavage, generating soluble fragments that are overexpressed in ADPKD kidneys. The regulation, localization, and function of these fragments is poorly understood. Here, we show that a ∼30 kDa cleavage fragment (PC1-p30), comprising the entire C-terminal tail, undergoes rapid proteasomal degradation by a mechanism involving the von Hippel-Lindau tumor suppressor protein. PC1-p30 is stabilized by reactive oxygen species, and the subcellular localization is regulated by reactive oxygen species in a dose-dependent manner. We found that a second, ∼15 kDa fragment (PC1-p15), is generated by caspase cleavage at a conserved site (Asp-4195) on the PC1 C-terminal tail. PC1-p15 is not subject to degradation and constitutively localizes to the mitochondrial matrix. Both cleavage fragments induce mitochondrial fragmentation, and PC1-p15 expression causes impaired fatty acid oxidation and increased lactate production, indicative of a Warburg-like phenotype. Endogenous PC1 tail fragments accumulate in renal cyst-lining cells in a mouse model of PKD. Collectively, these results identify novel mechanisms regarding the regulation and function of PC1 and suggest that C-terminal PC1 fragments may be involved in the mitochondrial and metabolic abnormalities observed in ADPKD.

Autosomal dominant polycystic kidney disease (ADPKD) is one of the most common life-threatening genetic disorders and is characterized by the progressive growth of fluid-filled cysts in both kidneys, often leading to end-stage renal failure by the sixth decade of life (1). ADPKD is caused by mutations in either the PKD1 or PKD2 gene, encoding for polycystin-1 (PC1) and polycystin-2 (PC2), respectively. Mutations in PKD1 account for over 85% of cases (2). The exact function of PC1, as well as the mechanism by which genetic defects lead to cystogenesis, remain poorly understood (3).

Defects in PC1 appear to drive cyst growth due to the aberrant activation of signaling pathways that are normally inactive in healthy adult cells (1). ADPKD is also associated with metabolic abnormalities reminiscent of the Warburg effect in cancer cells, characterized by aerobic glycolysis and mitochondrial dysfunction. These abnormalities as well as the cystic microenvironment leads to high levels of reactive oxygen species (ROS) in the kidneys, which in turn promote disease progression (4). The exact mechanism of how these changes occur in response to PC1 dysfunction is unknown.

PC1 is a large, multipass transmembrane protein that contains an extracellular N-terminal region and a cytoplasmic C-terminal tail (PC1-CT) (5). We and others have previously reported that the PC1-CT can be processed by proteolytic cleavage at multiple sites, generating a ∼30 kDa fragment (PC1-p30), and a smaller ∼15 kDa fragment (PC1-p15), corresponding to the entire soluble C-terminal tail and the extreme end, respectively (6, 7, 8). Although the exact mechanism is unclear, previous studies have shown that PC1-p30 release from the C-terminal tail occurs upon cessation of renal tubular fluid flow, possibly in a γ-secretase-dependent manner (7, 9). The extreme end of the PC1-CT contains a coiled-coil domain (10), through which PC1 interacts with PC2 to induce cation-permeable currents (11).

Both PC1-p30 and PC1-p15 are overexpressed in kidneys of ADPKD patients and nonorthologous rodent models of PKD (6, 8, 12). The PC1-CT has been implicated in the regulation of numerous signaling pathways that regulate proliferation, inflammation, and apoptosis (8, 9, 12, 13, 14, 15, 16). We and others have previously shown that soluble PC1-p30 can localize to the nucleus, where it regulates gene expression by coactivation of STAT6- and STAT3-transcription factors, and regulation of β-catenin signaling (6, 8, 16, 17).

Since both PC1-p30 and PC1-p15 are overexpressed in ADPKD, and since PC1-p30 is known to activate signaling pathways involved in cyst growth, it is reasonable to assume that these proteolytic fragments may play important roles in the mechanisms underlying cyst formation and/or cyst growth in ADPKD. We therefore sought to investigate the regulation and function of PC1-p30 and PC1-p15 to elucidate their roles in ADPKD.

We show that PC1-p30 is rapidly degraded by a ubiquitin/proteasome-mediated mechanism, stabilized by ROS, and can differentially target to the mitochondria or nucleus, respectively, depending on the level of oxidative stress. Additionally, we found that the PC1-CT can be cleaved by caspases to generate PC1-p15, which constitutively targets mitochondria and is not subject to rapid degradation. Overexpression of these cleavage fragments leads to mitochondrial fragmentation and inhibits fatty acid catabolism, indicative of impaired mitochondrial function and adoption of a Warburg-like phenotype. Our results suggest that PC1 is involved in a mechanism that regulates cellular metabolism by modifying mitochondrial function in response to changes in oxidative stress and caspase activation.

## Results

### PC1-p30 undergoes rapid ubiquitination and proteasomal degradation

**Figure 1:**
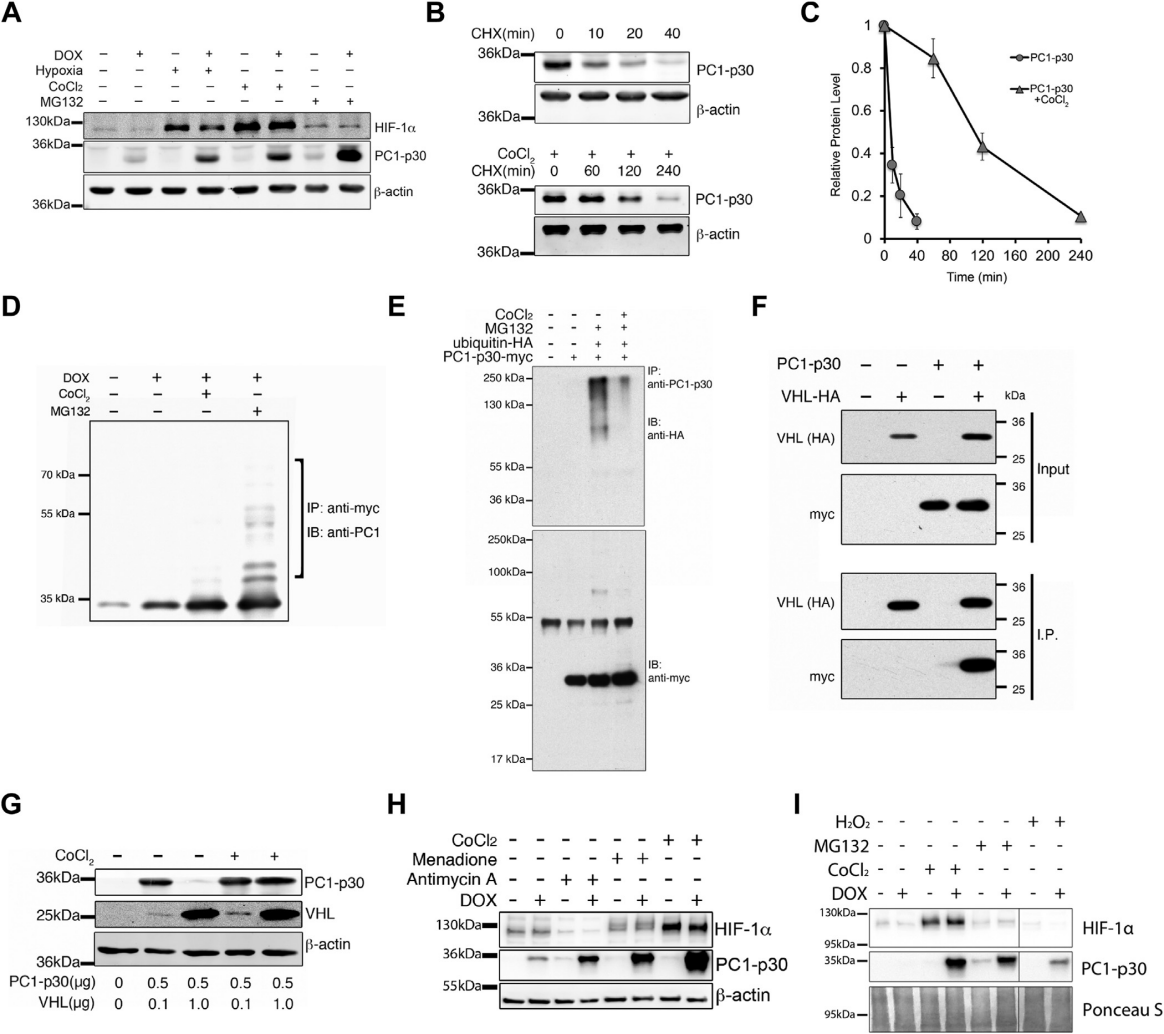
**PC1-p30 undergoes proteasomal degradation and is stabilized by ROS.***A*, immunoblot analysis of MDCK cells stably transfected with a DOX-inducible myc-tagged PC1-p30 construct (MDCK-p30). Cells were treated with DOX for 24 h and cultured in a hypoxia chamber (1% O_2_) for 16 h or treated with CoCl_2_ (250 μM) or MG132 (1.5 μM). *B*, estimated half-life of PC1-p30 by immunoblot in MDCK-p30 cells treated with the protein synthesis inhibitor cycloheximide (CHX) alone (*top panel*) or both CHX and CoCl_2_ (*bottom panel*). *C*, densitometry analysis of PC1-p30 immunoblot represented in *B*. Data presented as mean ± SD; n = 3 independent experiments. *D*, PC1-p30 was immunoprecipitated from MDCK-p30 cells using an anti-myc tag antibody. The ladder of higher molecular weight bands (*bracket*) in the presence of MG132 indicates poly-ubiquitination. *E*, coimmunoprecipitation from HEK293T cells transiently transfected with DOX-inducible PC1-p30-myc and HA-ubiquitin. PC1-p30 was pulled down using an antibody against the C terminus of PC1. The myc-reactive band at 55 kDa in the *lower panel* corresponds to the pull-down antibody heavy chain. *F*, coimmunoprecipitation from HEK293T cells transiently transfected with PC1-p30-myc and HA-tagged VHL. VHL was pulled down using an anti-HA antibody and PC1-p30 was visualized with an anti-myc antibody. *G*, immunoblot of HEK293T cells transiently transfected with different amounts of VHL or PC1-p30 cDNA and treated with CoCl_2_. *H*, immunoblot of PC1-p30 stabilization in MDCK-p30 cells treated with various ROS inducers: antimycin A (60 μM) and menadione (40 μM). *I*, immunoblot of PC1-p30 stabilization in MDCK-p30 cells treated with hydrogen peroxide (H_2_O_2_). DOX, doxycycline; ROS, reactive oxygen species; VHL, von Hippel-Lindau.

We set out to investigate the mechanism by which PC1-p30 expression is increased in kidneys from ADPKD patients (6, 7, 8, 12). In ADPKD, renal cyst expansion causes localized hypoxia, resulting in stabilization of hypoxia-inducible factor-1-alpha (HIF-1α), which is degraded by the ubiquitin-proteasome system under normal O_2_ levels (normoxia) (18, 19). Since PC1 was previously reported to be sensitive to cellular O_2_ levels (20), we hypothesized that in normoxia, PC1-p30 may be degraded by the ubiquitin-proteasome system, and that the increased abundance in ADPKD kidneys may be a result of the local hypoxic environment. Using MDCK cells stably transfected with a myc-tagged PC1-p30 construct under a doxycycline (DOX)-inducible promoter (MDCK-p30) (8), we found that PC1-p30 was indeed stabilized in hypoxia (1% O_2_ for 16 h) or following treatment with the hypoxia-mimetic cobalt chloride (CoCl_2_) or proteasome inhibitor MG132 (Fig. 1*A*). CoCl_2_ mimics hypoxia by replacing Fe^2+^ with Co^2+^ in prolyl hydroxylase (PHD) enzymes, oxygen-dependent sensors that hydroxylate proline residues on HIF-1α, resulting in its degradation (21). Fe^2+^ is a crucial cofactor in this reaction, and substituting Co^2+^ leads to PHD inhibition and resulting stabilization of HIF-1α (22).

To determine the half-life of PC1-p30, MDCK-p30 cells were treated with the protein synthesis inhibitor cycloheximide (CHX) alone (Fig. 1*B*, top panel) or in combination with CoCl_2_ (Fig. 1*B*, bottom panel). PC1-p30 exhibited a short half-life under 10 min, which was prolonged to 1 to 2 h by CoCl_2_ treatment (Fig. 1*C*). To determine the ubiquitylation status, PC1-p30 was immunoprecipitated from MDCK-p30 cells under denaturing conditions. Detection with an antibody against the C terminus of PC1 (anti-PC1-CT) revealed multiple higher molecular weight products following treatment with MG132, indicative of polyubiquitination (Fig. 1*D*). Coimmunoprecipitation of PC1-p30-myc and ubiquitin-hemagglutinin (HA) from HEK293T cells verified polyubiquitination of PC1-p30 following proteasomal inhibition with MG132, which was significantly reduced in the presence of CoCl_2_ (Fig. 1*E*). Together, these results indicate that PC1-p30 is rapidly degraded by the ubiquitin-proteasome system and that hypoxia inhibits this degradation.

### Degradation of PC1-p30 involves pVHL

Ubiquitination of HIF-1α requires binding to the von Hippel-Lindau tumor suppressor protein (pVHL), the substrate recognition component of a complex that possesses E3 ubiquitin ligase activity (23). Since PC1-p30 is stabilized in hypoxia, we reasoned that pVHL may enhance PC1-p30 degradation. Reciprocal coimmunoprecipitation from HEK293T cells transfected with PC1-p30-myc and pVHL-HA revealed that PC1-p30 and pVHL physically interact (Fig. 1*F*). pVHL overexpression dramatically reduced PC1-p30, which was restored by treatment with CoCl_2_ (Fig. 1*G*), indicating that interaction with pVHL promotes PC1-p30 degradation in normoxia.

HIF-1α association with pVHL is mediated by proline hydroxylation, which is carried out by the PHD enzymes (24). Since we had already found that CoCl_2_, a PHD inhibitor, results in PC1-p30 stabilization, we sought to determine if PC1-p30 stabilization can be induced by other PHD inhibitors. We treated MDCK-p30 cells with pan-PHD inhibitors roxadustat, L-mimosine, and dimethyloxalylglycine. Although roxadustat and L-mimosine stabilized HIF-1α, there was no effect on PC1-p30 stability (Fig. S1*A*). PC1-p30 contains several highly conserved proline residues (Fig. S1*B*), however, mutation of these residues did not inhibit PC1-p30 degradation (Fig. S1*C*). These results suggest that the mechanism of binding between pVHL and PC1-p30 is independent of proline hydroxylation.

### PC1-p30 is stabilized by ROS

Our finding that PC1-p30 stabilization is independent of proline hydroxylation led us to examine molecular events downstream of hypoxia. Hypoxia leads to the formation of ROS, which act as signaling molecules in the hypoxic response (25, 26). CoCl_2_ is also known to induce oxidative stress (27, 28). Therefore, to determine if ROS are sufficient to stabilize PC1-p30, we treated MDCK-p30 cells with several ROS-inducing compounds. Treatment with both menadione (a redox cycler) and antimycin A (a mitochondrial electron transport chain inhibitor) stabilized PC1-p30 (Fig. 1*H*), indicating that ROS inhibit its degradation. Additionally, we treated cells with hydrogen peroxide (H_2_O_2_) as an external source of ROS. H_2_O_2_ treatment resulted in PC1-p30 stabilization (Fig. 1*I*).

Together, these results suggest that in normoxia when cellular ROS levels are low, PC1-p30 undergoes rapid ubiquitination and proteasomal degradation in a pVHL-dependent manner and is stabilized by ROS generated in hypoxia-dependent or hypoxia-independent mechanisms.

### PC1-p30 subcellular localization is regulated by ROS levels and induces mitochondrial fragmentation

**Figure 2:**
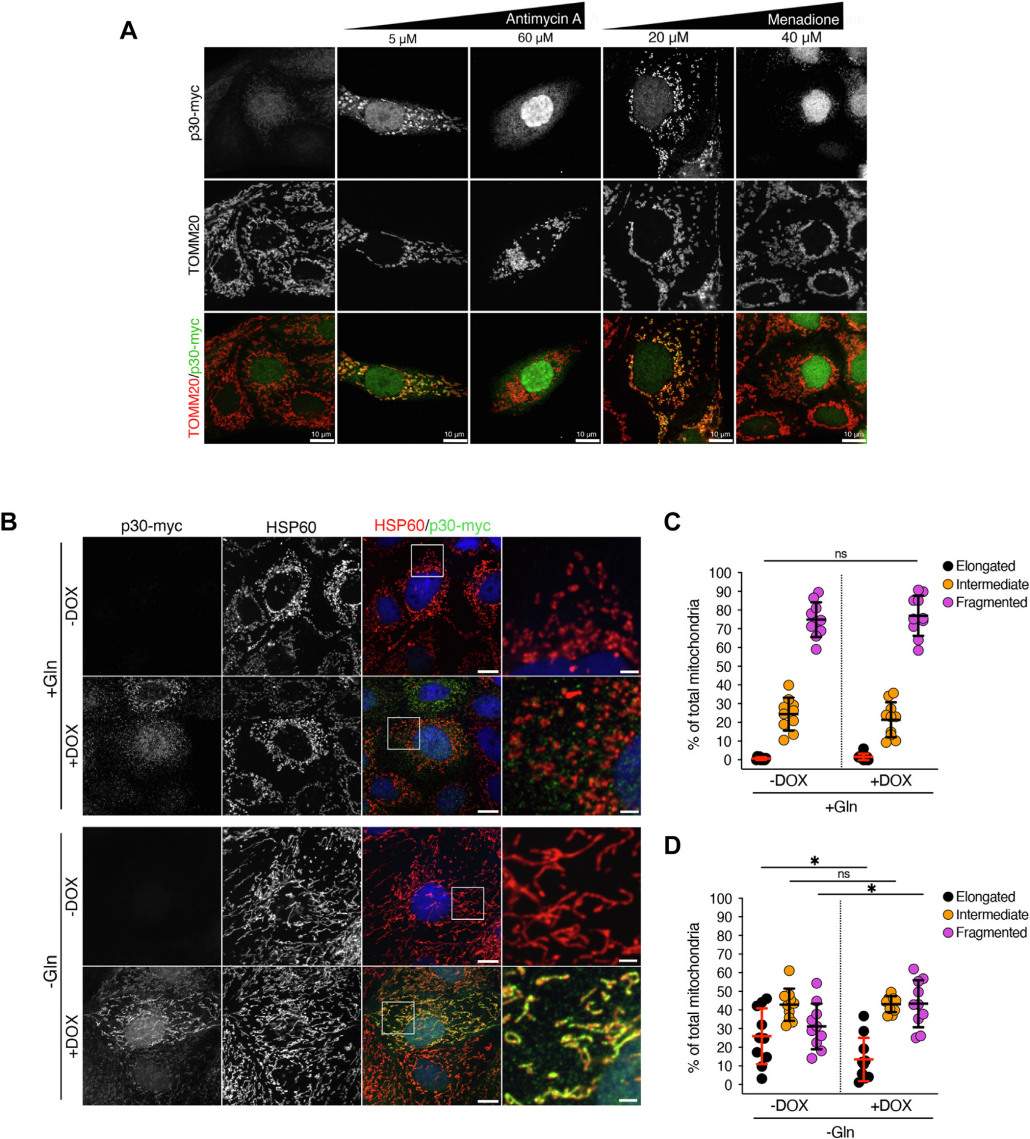
**PC1-p30 subcellular localization is regulated by ROS levels and induces mitochondrial fragmentation.***A*, differential subcellular targeting of PC1-p30 was observed by immunofluorescence staining for myc-tag (PC1-p30) and TOMM20 (mitochondrial marker) in MDCK-p30 cells, following treatment with various concentrations of ROS inducers. *B*, representative immunofluorescence images of MDCK-p30 cells cultured in complete media supplemented with (+Gln) or without (-Gln) 2 mM L-glutamine for 48 h. Cells were stained with anti-myc-tag (PC1-p30) and anti-HSP60 (mitochondrial marker) antibodies. Nuclei were counterstained with DAPI. *C* and *D*, mitochondrial morphology quantification of images represented in *B*. Individual mitochondria were classified into three distinct morphological classes: elongated, intermediate, or fragmented, and the percentage of each class was normalized to the total amount of mitochondria per cell. Each data point represents one cell, with n = 50 cells/condition. The scale bars in overview images represent 10 μm. The scale bars in insets represent 2 μm. Data is presented as the mean ± SD (∗*p* < 0.05, ∗∗*p* < 0.01, ∗∗∗*p* < 0.001, and ∗∗∗∗*p* < 0.0001). ROS, reactive oxygen species.

We and others have shown that PC1-p30 exhibits nuclear and cytoplasmic localization (7, 8, 9, 12, 16), and a previous study reported that PC1-p30 can localize to mitochondria (29). We therefore set out to determine where PC1-p30 localizes in response to ROS inducers. We found that PC1-p30 localized to mitochondria in MDCK-p30 cells treated with low concentrations of antimycin A or menadione (Fig. 2*A*). In contrast, high concentrations of both compounds induced strong nuclear accumulation of PC1-p30 (Fig. 2*A*), indicating that ROS regulates differential targeting of PC1-p30 to the mitochondria or nucleus, respectively, in a dose-dependent manner.

The observed mitochondrial targeting prompted us to investigate whether PC1-p30 affects mitochondrial morphology. While mitochondria regularly undergo fission and fusion events, an imbalance toward increased fission leading to small, fragmented mitochondria is associated with mitochondrial dysfunction (30). In normal culture conditions MDCK cells are highly glycolytic (31) and exhibit fragmented mitochondria, potentially masking additional changes to the mitochondrial network. Culturing MDCK-p30 cells in the absence of L-glutamine (L-Gln) for 48 h led to mitochondrial elongation (Fig. 2*B*), similar to what has been reported for other cell lines (32, 33). Since L-Gln deprivation induces ROS due to depletion of the antioxidant GSH (34), PC1-p30 is localized to mitochondria under these conditions (Fig. 2*B*). Quantification of mitochondrial morphology in MDCK-p30 cells under normal (+Gln) and L-Gln deprivation (-Gln) conditions revealed that in the absence of L-Gln, PC1-p30 expression led to a higher percentage of fragmented mitochondria, but had no effect in culture conditions where L-Gln was supplied (Fig. 2, *B*–*D*), suggesting that mitochondrially targeted PC1-p30 regulates mitochondrial morphology.

### PC1 C-terminal fragments are overexpressed in cyst-lining cells *in vivo*

**Figure 3:**
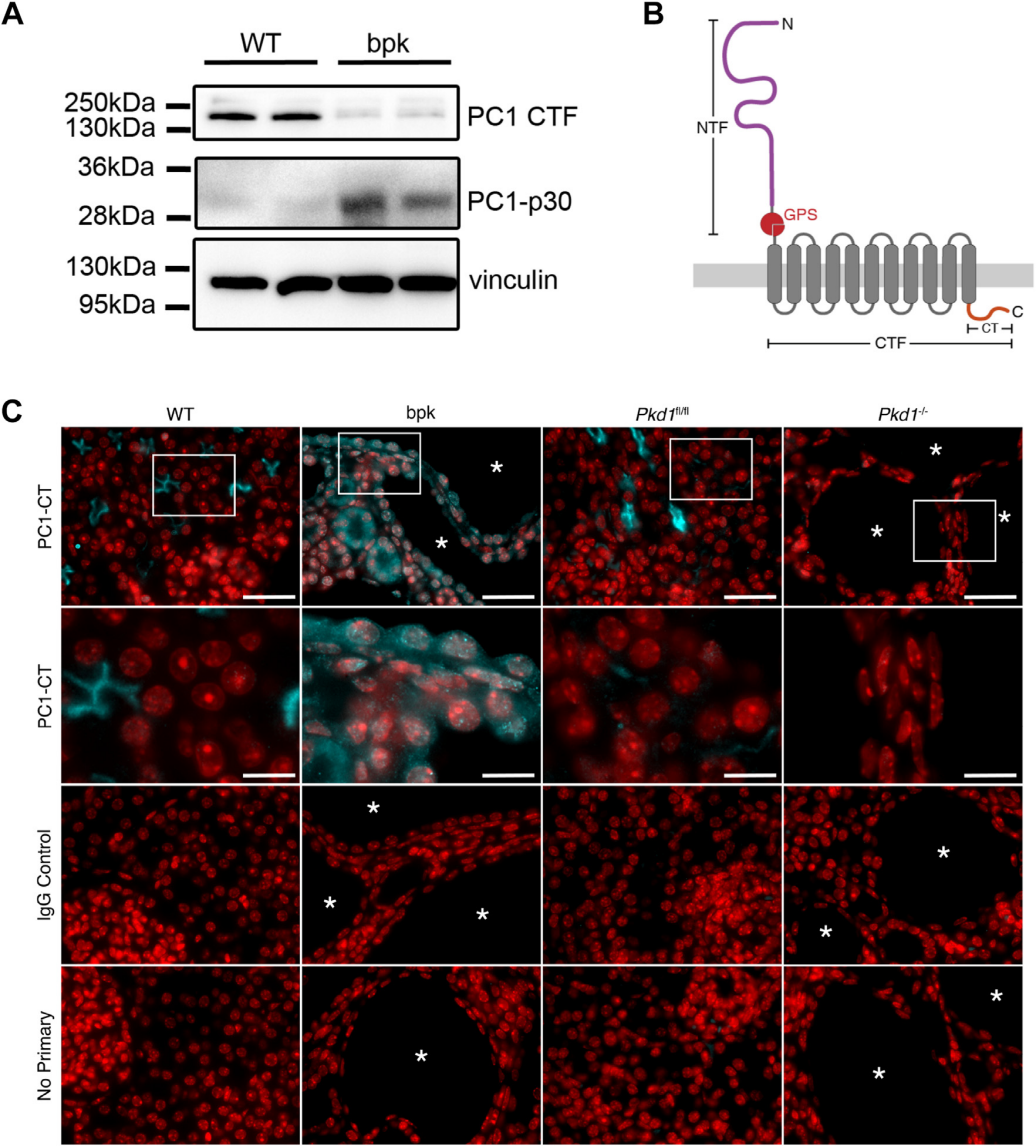
**PC1 C-terminal fragments are overexpressed in cyst-lining cells *in vivo.****A*, immunoblot of whole kidney lysates from age-matched WT and cystic *bpk* mice using an antibody against the C-terminal tail of PC1 (PC1-CT). The band at 150 kDa corresponds to PC1-CTF, and the band at 30 kDa corresponds to PC1-p30. Vinculin was used as a loading control. *B*, schematic of full-length PC1 and corresponding cleavage sites (NTF, N-terminal fragment resulting from GPS cleavage; CTF, C-terminal fragment resulting from GPS cleavage; CT, C-terminal tail.) *C*, immunofluorescence staining of kidney sections from WT, *bpk*, *Pkd1*^*fl/fl*^, and *Pkd1*^*−/−*^ (*Pkd1*-tamoxifen inducible KO) mice. Sections were stained with the same mouse anti-PC1-CT antibody used in A or a mouse IgG isotype control (*cyan*). Nuclei were counterstained with DAPI (*red*). Cysts are labeled with *asterisks* (∗). The *second row* are zoomed-in insets marked by *white rectangles* in the *top row*. The scale bars in overview images represents 20 μm. The scale bars in insets represent 7 μm. bpk, Balb/c polycystic kidney; PC1-CTF, PC1 C-terminal fragment.

Endogenous PC1 is notoriously difficult to detect, and its subcellular localization in both healthy and cystic kidneys is still debated (35). To detect PC1-p30 *in vivo*, we used the Balb/c polycystic kidney (*bpk*) mouse model, a nonorthologous model for early-onset PKD in which the *Pkd1* alleles are unaffected (36). Due to the rapid cyst growth in these mice, we hypothesized that PC1-p30 would be stabilized in cyst-lining cells with high levels of ROS due to the local hypoxic environment. We generated a novel mouse mAb against the PC1 C-terminal tail that is reactive to both the human and mouse protein. We detected a ∼30 kDa band in kidney lysates from *bpk* mice, but not in age-matched WT controls (Fig. 3*A*). Conversely, a ∼150 kDa band was detected only in WT mice, corresponding to the PC1 C-terminal fragment (PC1-CTF) that is generated during PC1 maturation by cleavage of full-length PC1 at the G protein-coupled receptor proteolytic site cleavage site (Fig. 3, *A* and *B*) (35). The PC1-CTF band was reduced in *bpk* mice, correlating with the increased abundance of the PC1-p30 band. These results suggest that in healthy kidneys, the C-terminal tail remains associated with membrane-anchored CTF but is cleaved from PC1-CTF in cystic kidneys.

**Figure 4:**
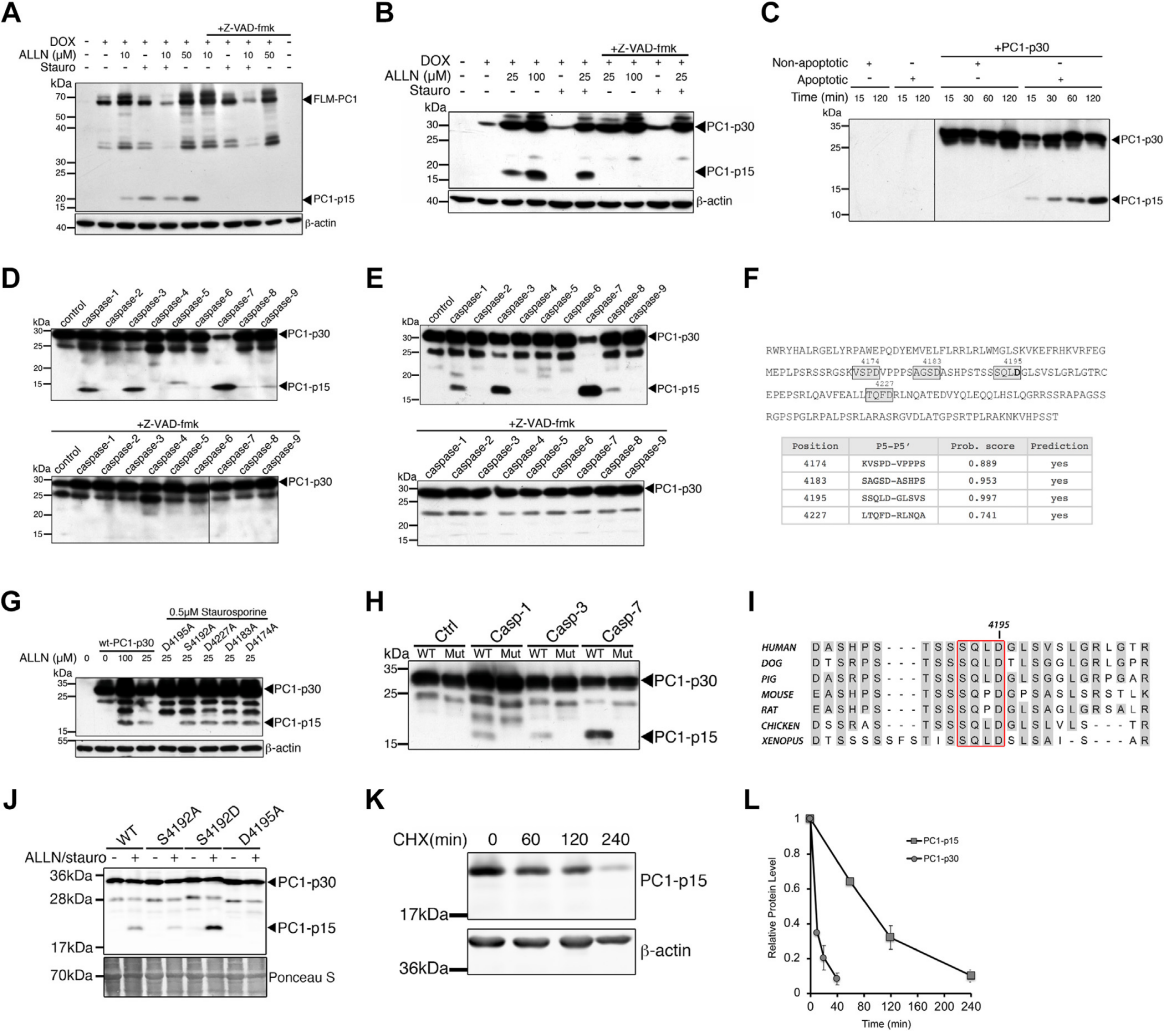
**PC1-p15 is generated by caspase-dependent cleavage of PC1 at Asp-4195.***A* and *B*, immunoblots of MDCK cells expressing DOX-inducible full-length membrane-bound PC1 (PC1-FLM) (*A*) or soluble PC1-p30 (*B*) constructs treated with apoptosis-inducers ALLN or staurosporine and the pan-caspase inhibitor z-VAD-fmk. *C*, immunoblot of bacterially expressed, purified PC1-p30 was incubated with untreated (nonapoptotic) or staurosporine-treated (apoptotic) cytosol from MDCK cells. PC1-p15 migrates at a lower molecular weight without the myc2-tag. *D* and *E*, immunoblot analysis of PC1-p15 cleavage by recombinant purified caspases 1 to 9 from bacterially expressed, purified PC1-p30 (*D*) or HEK293T cells expressing myc2-His6-tagged PC1-p30 (*E*). *F*, human PC1-p30 sequence with predicted caspase cleavage sites (*boxes*) with high-probability scores (*table*). *G*, immunoblot of MDCK cells expressing either WT PC1-p30 or PC1-p30 noncleavable mutants with *D* to *A* substitutions in predicted cleavage sites. *H*, immunoblot of MDCK cells expressing WT PC1-p30 (WT) or the D4195A mutant construct (Mut) and incubated with recombinant caspases. *I*, aligned sequences surrounding the cleavage site at Asp-4195 of PC1-p30. The tetrapeptide recognition sequence is outlined in *red*. *J*, immunoblot of HEK293T cells expressing WT-PC1-p30, a nonphosphorylatable (S4192A), a phospho-mimetic (S4192D) or cleavage (D4195A) mutation constructs. Ponceau S staining shown as loading control. *K*, immunoblot analysis of PC1-p15 half-life in MDCK-p15 cells following treatment with CHX. *L*, densitometry analysis of PC1-p15 blot represented in K. PC1-p30 is shown for comparison (quantification in Fig. 1*B*). Data is presented as the mean ± S.D.; n = 3 independent experiments. CHX, cycloheximide; ROS, reactive oxygen species.

We then performed immunofluorescence staining of kidney sections from WT and *bpk* mice with the same antibody used in Figure 4*A*. We detected a signal on the apical membrane of some tubules in WT mice but found prominent cytoplasmic and nuclear staining in cysts and tubules in *bpk* mice (Fig. 3*C*). Nuclear and cytoplasmic accumulation was observed in cyst-lining epithelium of *bpk* mice, indicating the presence of cleaved, soluble PC1-p30. To confirm antibody specificity, we used a tamoxifen-inducible homozygous *Pkd1* KO mouse model that has been previously described (*Pkd1*^*fl/fl*^*;* uninduced WT, *Pkd1*^*−/−*^*;* induced KO) (37). In *Pkd1*^*fl/fl*^ mice, PC1 staining was consistent with the staining pattern observed in WT mice. No PC1 signal was observed in *Pkd1*^*−/−*^ mice, or in any kidney sections probed with a nonspecific mouse immunoglobulin G (IgG), indicating that the PC1 tail antibody is specific to PC1. These results indicate that PC1-p30 accumulates in cyst-lining cells *in vivo* in the *bpk* mouse model.

### PC1-p15 is generated by caspase-dependent cleavage of PC1 at Asp-4195

We had previously discovered a ∼15 kDa PC1 C-terminal fragment that accumulates alongside PC1-p30 in human ADPKD kidneys (6). In addition, we previously showed that a ∼15 kDa cleavage fragment of the PC1-CT (PC1-p15) can be generated from a membrane-anchored PC1 tail construct (full-length membrane-bound PC1 [FLM-PC1]), following treatment with the proteasome inhibitor ALLN (8).

Consistent with previous results, PC1-p15 was detected in MDCK cells stably transfected with a DOX-inducible FLM-PC1 construct, following treatment with ALLN (Fig. 4*A*). We observed significant cell death with higher concentrations of ALLN, which led to more PC1-p15 cleavage, suggesting that apoptotic stimuli may be responsible. Therefore, we treated cells with the apoptosis-inducer staurosporine, which led to PC1-p15 cleavage that was abolished by the caspase inhibitor z-VAD-fmk (Fig. 4*A*). PC1-p15 was also detected in MDCK-p30 cells in the presence of ALLN or staurosporine, indicating that PC1-p15 can also be generated from soluble PC1-p30 (Fig. 4*B*). To further investigate cleavage of PC1, we carried out an *in vitro* cleavage assay of bacterially expressed, purified PC1-p30 incubated with cytosol extracted from untreated (nonapoptotic) or staurosporine-treated (apoptotic) MDCK cells. Apoptotic cytosol caused time-progressive cleavage of PC1-p15 from PC1-p30 (Fig. 4*C*). Many caspases are primary effectors of apoptosis. To determine if caspases are responsible for PC1-p15 cleavage, we incubated PC1-p30 with a panel of purified, active caspases. PC1-p15 was cleaved by caspase-1, caspase-3, and caspase-7 from purified PC1-p30 produced in a bacterial (Fig. 4*D*) or mammalian cell system (Fig. 4*E*).

Caspases generally recognize their substrates at tetrapeptide cleavage motifs ending with an aspartate residue (38, 39). Using the CaspDB database (39), we identified four putative cleavage sites that would generate a fragment of approximately 15 kDa (Fig. 4*F*). We generated uncleavable mutation constructs of each site by replacing the aspartate residues with alanine and found that only the D4195A mutation prevented cleavage (Fig. 4*G*). Incubation of the D4195A construct with purified caspase-1, caspase-3, and caspase-7 abolished cleavage (Fig. 4*H*).

Caspase cleavage can be influenced by phosphorylation of nearby serine residues (40, 41). PC1 contains a highly conserved serine residue upstream of Asp-4195 (Fig. 4*I*), suggesting that phosphorylation may regulate caspase-dependent cleavage. Expression of a nonphosphorylatable mutant (S4192A) decreased PC1-p15 cleavage, whereas a phospho-mimetic mutant (S4192D) increased cleavage (Fig. 4*J*).

We generated MDCK cells stably transfected with a myc-tagged PC1-p15 construct corresponding to aa 4196 to 4303 of human PC1 under the control of a DOX-inducible promoter (MDCK-p15). Treatment with CHX revealed that PC1-p15 has a long half-life of over 1.5 h (Fig. 4*K*), indicating that PC1-p15, unlike PC1-p30, is not rapidly degraded (Fig. 4*L*). Together, these results indicate that PC1-p15 is a relatively stable protein fragment generated by caspase-1–, caspase-3–, or caspase-7–mediated cleavage at Asp-4195 from both membrane-anchored PC1 and soluble PC1-p30.

While we successfully detected PC1-p30 in cystic mice, we were unable to detect PC1-p15 in kidney lysates from either WT or *bpk* mice. In rodents, the tetrapeptide caspase recognition motif contains a proline residue as opposed to leucine in humans (Fig. 4*I*), and we wanted to test whether this substitution in rodents dampens or inhibits cleavage. We found that PC1-p15 was effectively cleaved in cells transfected with a mouse PC1-p30 construct (Fig. S3), indicating that this motif is subject to cleavage in rodents, humans, and presumably other species. We speculate that we were unable to detect PC1-p15 due to a low level of caspase activity in this mouse model.

### The PC1 C-terminal tail contains a ROS-responsive motif and a mitochondrial targeting sequence

**Figure 5:**
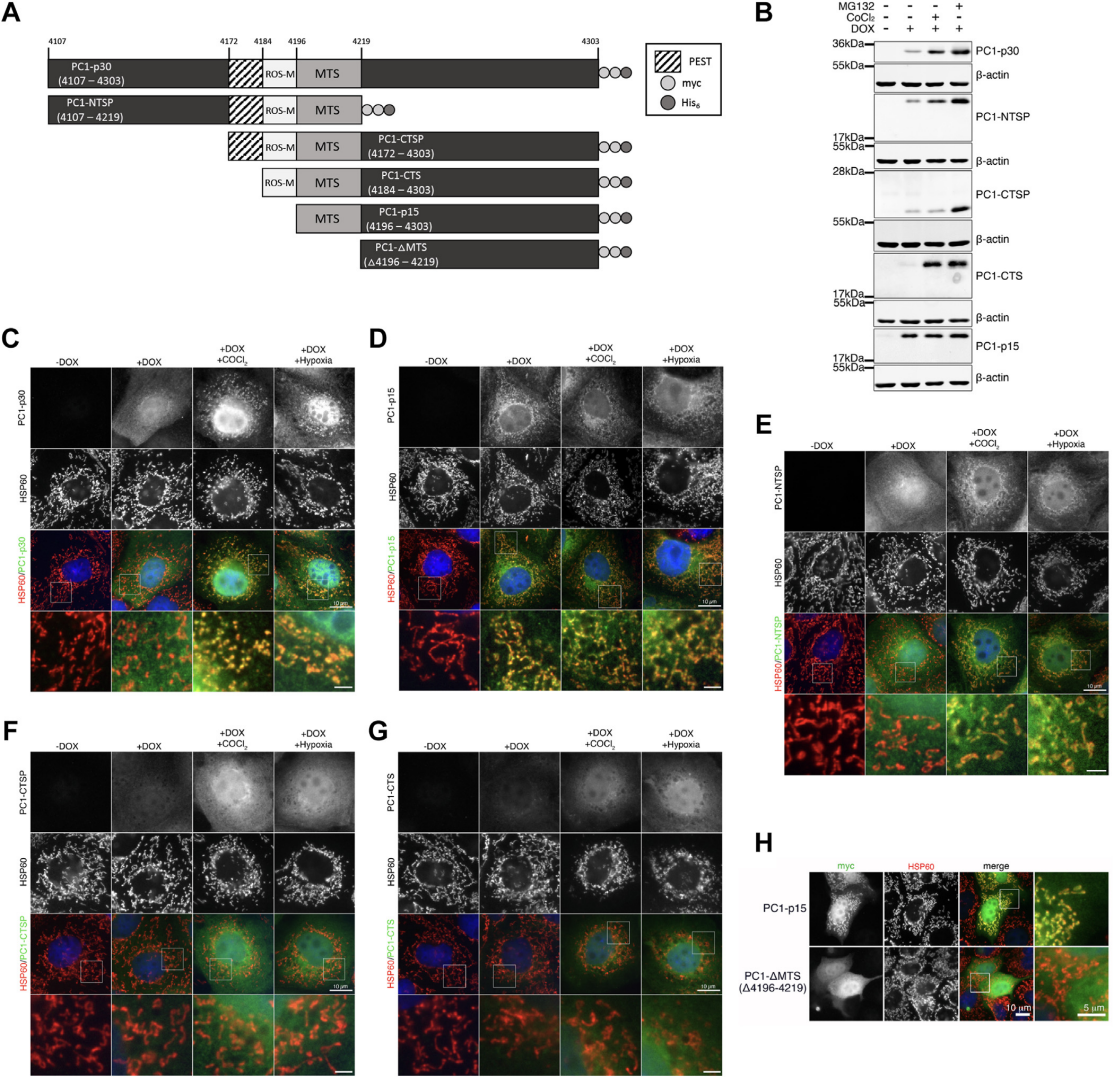
**The PC1 C-terminal tail contains a ROS-responsive motif and a mitochondrial targeting sequence.***A*, schematic of PC1 C-terminal tail (PC1-CTT) constructs used to generate DOX-inducible stable cell lines used in B to H. *B*, immunoblot of MDCK stable cell lines expressing PC1-CTT constructs following treatment with MG132 (1.5 μM) or CoCl_2_ (200 μM). *C*–*G*, immunofluorescence staining for myc-tag (PC1 constructs) and HSP60 (mitochondrial marker) in MDCK cell lines expressing PC1-CTT constructs: (*C*) PC1-p30, (*D*) PC1-p15, (*E*) PC1-NTSP, (*F*) PC1-CTSP, or (*G*) PC1-CTS following treatment with CoCl_2_ or subjected to hypoxia (1% O_2_) for 16 h. *H*, immunofluorescence staining of MDCK cells expressing WT PC1-p15 or a PC1-p15 mutation construct containing a deletion within the predicted mitochondrial targeting sequence (PC1-ΔMTS). The scale bars in overview images represent 10 μm. The scale bars in insets of 5C/D/E/G represent 2 μm. The scale bar in inset of 5H represent 5 μm. MTS, mitochondrial targeting sequence; ROS-M, ROS-dependent stabilization motif.

Knowing that PC1-p15 has a longer half-life than PC1-p30, we set out to determine characteristic regions of the PC1 tail responsible for the ROS-dependent stabilization and mitochondrial targeting of PC1-p30. We generated stable MDCK cell lines expressing DOX-inducible constructs of the PC1 C-terminal tail (Fig. 5*A*). PC1-p30, PC1-NTSP, PC1-CTSP, and PC1-CTS were all stabilized following treatment with MG132 (Fig. 5*B*). Only PC1-p30, PC1-NTSP, and PC1-CTS were stabilized in response to CoCl_2_ treatment, indicating that these constructs share a region that enables ROS-dependent stabilization. The inability of PC1-CTSP to be stabilized by CoCl_2_ may be due to the presence of an exposed N-terminal PEST sequence, which we previously found promotes degradation (8). PC1-p15 was unaffected by CoCl_2_, suggesting the putative ROS-dependent stabilization region is upstream of aa 4196. Excluding this downstream region, the only common region between PC1-p30, PC1-NTSP, and PC1-CTS is between aa 4184 to 4195, suggesting that this region contains a ROS-dependent stabilization motif (ROS-M, Figures 5*A* and S2).

Immunofluorescence staining of MDCK-p30 showed that PC1-p30 localizes to the mitochondria when cells were cultured under hypoxic conditions (Fig. 5*C*). In contrast, PC1-p15 constitutively localized to mitochondria (Fig. 5*D*), suggesting that a mitochondrial targeting signal (MTS) is located between aa 4196 to 4303 of the PC1 tail. The subcellular localization of PC1-NTSP was the same as PC1-p30, localizing to mitochondria only under hypoxia (Fig. 5*E*), indicating that the MTS resides between aa 4196 to 4219, the only region of overlap between PC1-p30, PC1-p15, and PC1-NTSP (Fig. 5*A*). Neither PC1-CTSP nor PC1-CTS is localized to mitochondria (Fig. 5, *F* and *G*), suggesting that the region between aa 4172 to 4184 may mask the MTS. Deletion of the putative MTS (aa 4196–4219) abolished mitochondrial localization (Fig. 5*H*), indicating this region is responsible for mitochondrial targeting.

### Cleavage of the PC1 tail abrogates STAT signaling but PC1-p15 can still interact with PC2

**Figure 6:**
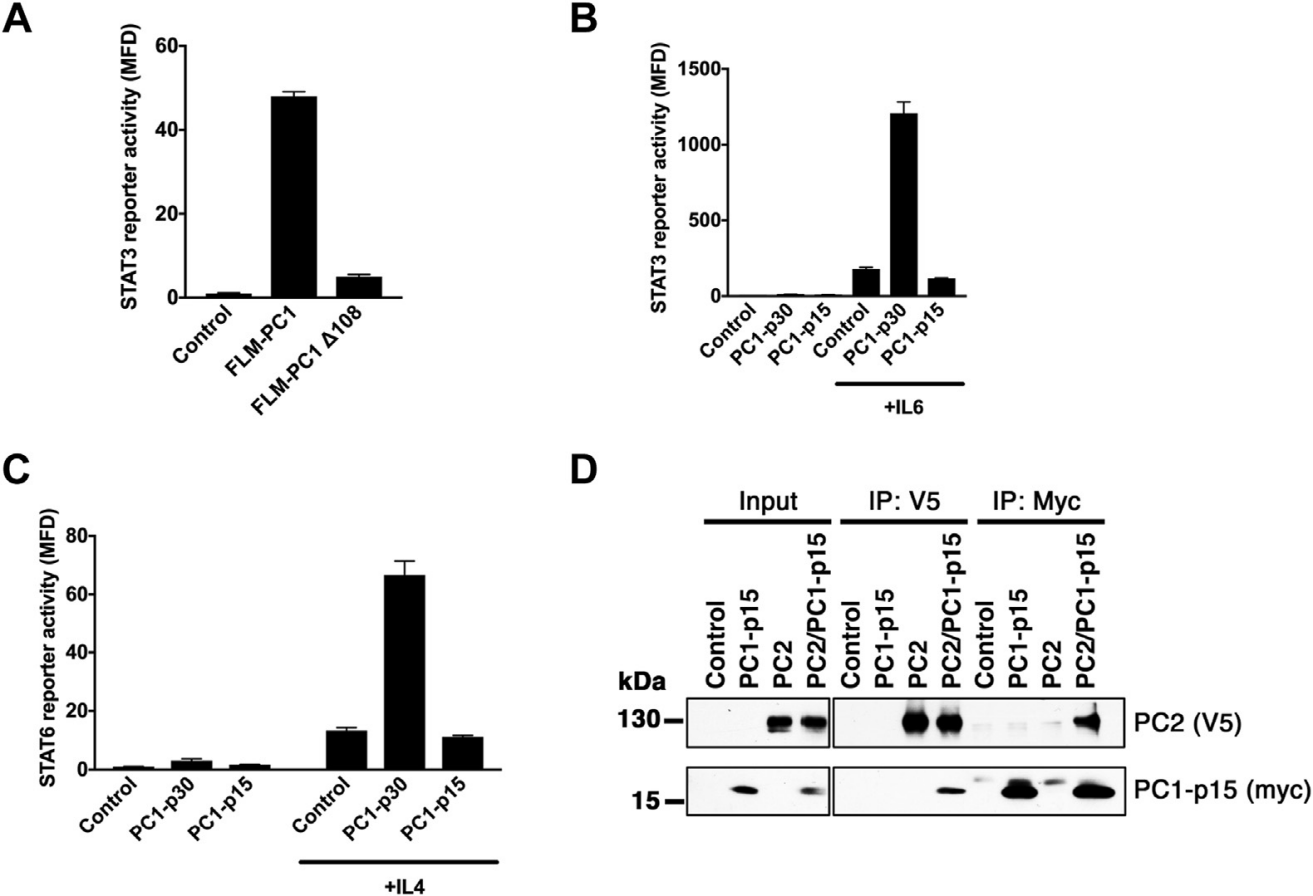
**Cleavage of the PC1 tail abrogates STAT signaling but PC1-p15 can still interact with PC2.***A*, STAT3 activity assay using HEK293T cells transfected with a STAT3 luciferase reporter and either FLM-PC1 or FLM-PC1 truncated at D4195 (FLM-PC1D108). *B* and *C*, STAT activity assays in HEK293T cells transfected with PC1-p30, PC1-p15, or empty vector control and (*B*) STAT3 or (*C*) STAT6 luciferase reporters and incubated with the indicated cytokines. *D*, coimmunoprecipitation of PC2 (V5-tag) and PC1-p15 (C-terminal myc-tag) from transiently transfected HEK293T cells. Data is presented as the mean ± SD. FLM-PC1, full-length membrane-bound PC1; STAT, signal transducer and activator of transcription.

We previously reported that the cytoplasmic tail of FLM-PC1 can activate signal transducer and activator of transcription 3 (STAT3), and that soluble PC1-p30 can coactivate STAT3 and STAT6 in the presence of interleukin-6 (IL6) or interleukin-4 (IL4), respectively (6, 8, 16). Using a STAT3 luciferase reporter assay, we tested whether cleavage at the Asp-4195 site would affect STAT3 activation by FLM-PC1. Truncation of the PC1 tail at the caspase cleavage site (FLM-PC1 Δ108) abolished STAT3 activation by the membrane-anchored PC1 tail (Fig. 6*A*). PC1-p15 had no effect on either STAT3 or STAT6 activity (Fig. 6, *B* and *C*). These results indicate that cleavage abrogates the ability of both the membrane-anchored and soluble PC1-CT to regulate STAT signaling.

PC1-p15 contains a coiled-coil domain that is critical for the interaction between PC1 and PC2 (10, 42). To determine if PC1-p15 is capable of binding to PC2, we transiently expressed myc-tagged PC1-p15 (PC1-p15-myc) and V5-tagged PC2 (PC2-V5) in HEK293T cells and performed a coimmunoprecipitation assay (Fig. 6*D*). Binding between PC1-p15 and PC2 was detected following immunoprecipitation with antibodies against either protein, indicating that the coiled-coil domain within PC1-p15 is sufficient for binding to PC2.

### PC1-p15 induces mitochondrial fragmentation

**Figure 7:**
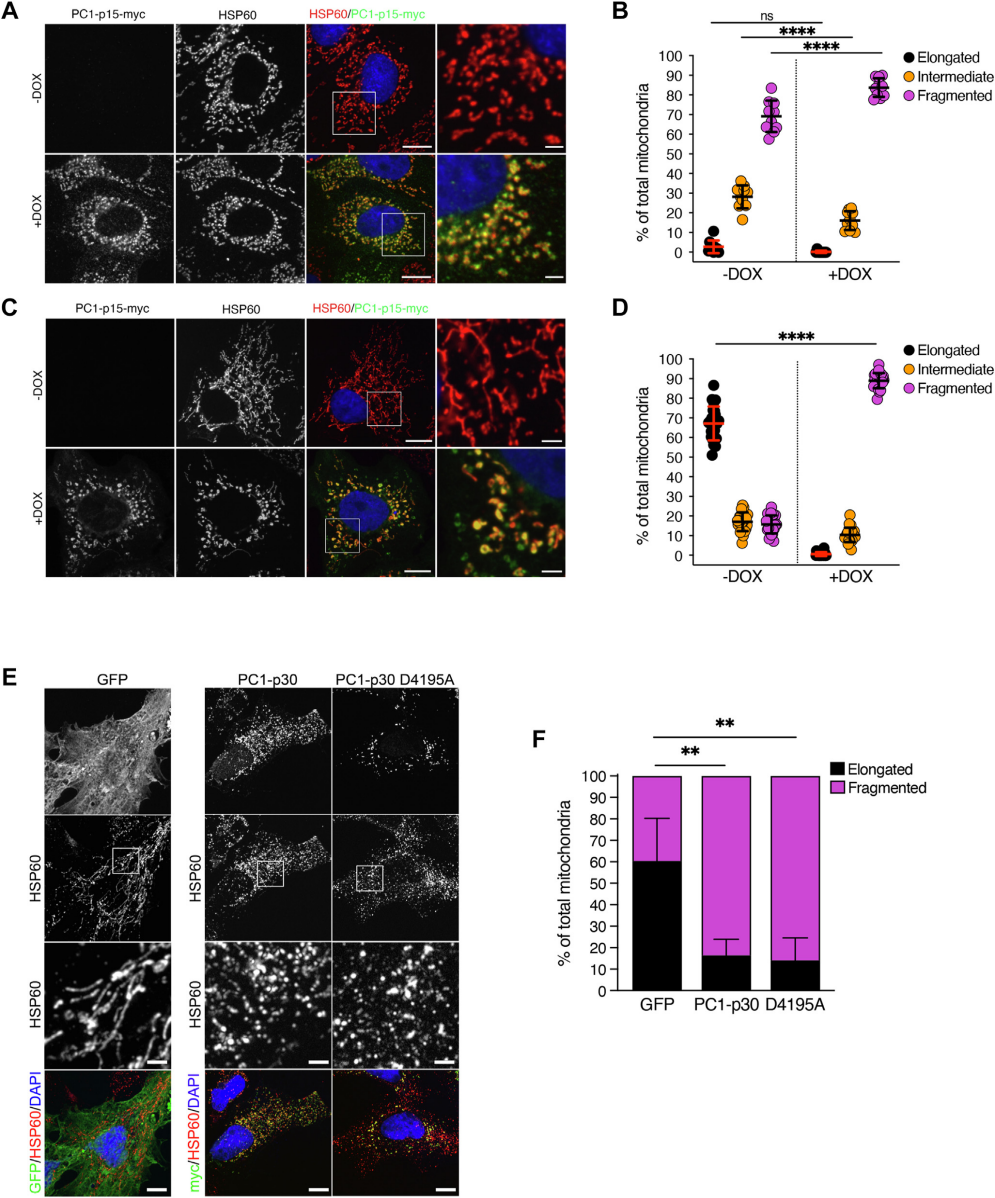
**PC1-p15 induces mitochondrial fragmentation.***A*–*D*, mitochondrial morphology quantification in stable cell lines expressing a DOX-inducible PC1-p15 construct. MDCK-p15 (*A* and *B*) or OK-p15 (*C* and *D*) cells were costained with anti-myc-tag (PC1-p15) and anti-HSP60 (mitochondria) antibodies and individual mitochondria were classified as either elongated, intermediate, or fragmented. Each data point represents one image, n = 50 cells/condition. *E*, immunofluorescence staining of OK cells transfected with GFP (control), PC1-p30, or a noncleavable (D4195A) PC1-p30 construct. *F*, mitochondrial morphology quantification of images represented in *E*. Since transient transfection already induces mitochondrial fragmentation, individual mitochondria were classified as either elongated or fragmented, n = 5 cells/condition. The scale bars in overview images represent 10 μm. The scale bars in insets represent 2 μm. Data is presented as the mean ± SD (∗*p* < 0.05, ∗∗*p* < 0.01, ∗∗∗*p* < 0.001, and ∗∗∗∗*p* < 0.0001). DOX, doxycycline; OK, opossum kidney.

To determine if PC1-p15 also affects mitochondria, we quantified mitochondrial morphology in MDCK-p15 cells (Fig. 7, *A* and *B*) and found that PC1-p15 expression induced mitochondrial fragmentation. Since MDCK cell mitochondria are very fragmented to begin with, we were interested in using a model with a more elongated mitochondrial phenotype. We therefore generated OK (opossum kidney) cells stably expressing myc-tagged PC1-p15 under a DOX-inducible promoter (OK-p15). Unlike MDCK cells, OK cells are derived from proximal tubule cells, which rely heavily on oxidative phosphorylation for ATP production to meet their high energy demand (43). Mitochondrial density and network elongation are crucial for metabolic function in these cells, making them an ideal model to study alterations in mitochondrial morphology (44). Cells not expressing PC1-p15 exhibited elongated mitochondria, while PC1-p15 expression led to a strong increase in mitochondrial fragmentation (Fig. 7, *C* and *D*).

Since both PC1-p30 and PC1-p15 induce mitochondrial fragmentation (Figs. 2*D* and 7, B/*D*, respectively), and since PC1-p15 can be cleaved from PC1-p30 (Fig. 4*B*), we investigated the possibility that the mitochondrial fragmentation induced by PC1-p30 may, in fact, be due to conversion of PC1-p30 into PC1-p15 followed by its effect on mitochondria. OK cells were transiently transfected with either control GFP, PC1-p30, or the uncleavable mutant of PC1-p30 (PC1-p30 D4195A). Compared to control GFP expression, both PC1-p30 and the uncleavable mutant induced mitochondrial fragmentation to the same extent (Fig. 7, E/*F*). This indicates that PC1-p30–induced mitochondrial fragmentation is not due to cleavage into PC1-p15.

### PC1-p15 is localized in the mitochondrial matrix and requires mitochondrial localization to induce fragmentation

**Figure 8:**
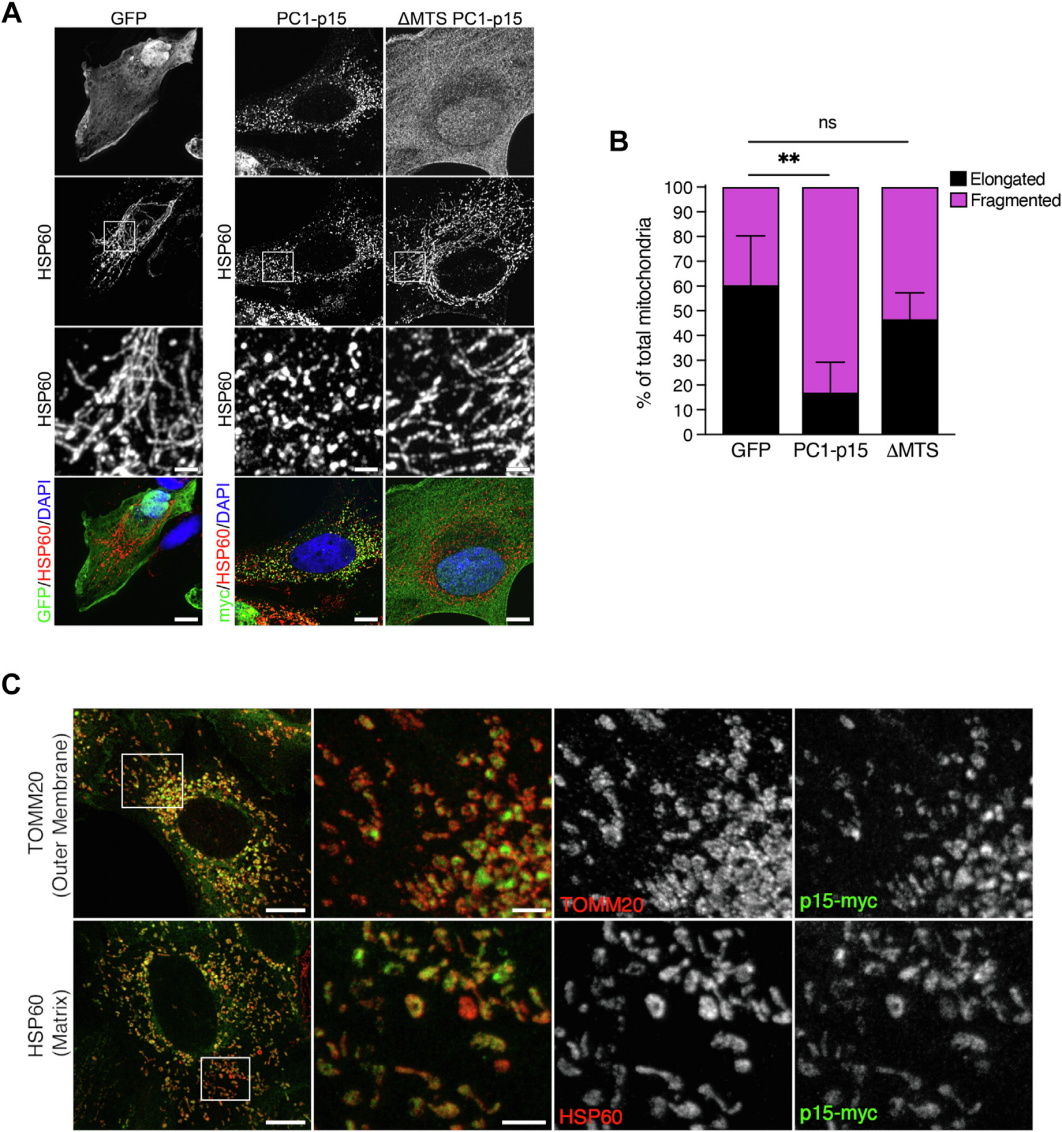
**PC1-p15 is localized in the mitochondrial matrix and requires a mitochondrial targeting sequence to induce fragmentation.***A*, immunofluorescence staining of OK cells transiently transfected with GFP (control), PC1-p15, or ΔMTS-PC1-p15 (MTS deletion mutant) constructs. *B*, mitochondrial morphology quantification of images represented in *A*. Individual mitochondria were classified as either elongated or fragmented. Data is presented as the mean ± SD, n = 5 cells/condition (∗*p* < 0.05, ∗∗*p* < 0.01, ∗∗∗*p* < 0.001, and ∗∗∗∗*p* < 0.0001). *C*, stimulated emission depletion (STED) super-resolution microscopy images of DOX-induced OK-p15 cells costained for PC1-p15 (p15-myc) and either HSP60 (mitochondrial matrix marker) or TOMM20 (mitochondrial outer membrane marker). *White boxes* on confocal overview (*left-most column*) indicate regions imaged with STED microscopy. Overlayed STED images (*second column*) show that PC1-p15 is enclosed by TOMM20 (*top*) and colocalizes with HSP60 (*bottom*). The scale bars in *A* and *C* confocal overview images represent 10 μm. The scale bars in STED images represent 2 μm. The scale bars in *A* insets represent 2 μm. DOX, doxycycline; MTS, mitochondrial targeting signal; OK, opossum kidney.

Having determined that PC1-p15 potently induces mitochondrial fragmentation, we set out to determine whether mitochondrial localization is necessary for fragmentation to occur. We transiently transfected OK cells with control GFP, PC1-p15, or the ΔMTS PC1-p15 mutant, which lacks the mitochondrial targeting sequence. The ΔMTS PC1-p15 mutant failed to fragment mitochondria (Fig. 8, A/*B*), suggesting that mitochondrial localization is required for the effect of PC1-p15.

To determine the exact location of PC1-p15 on mitochondria we used stimulated emission depletion super-resolution microscopy to image OK-p15 cells costained with PC1-p15 and with either TOMM20, an integral outer mitochondrial membrane protein, or HSP60, a mitochondrial matrix chaperone. We found that PC1-15 is enclosed by TOMM20, but colocalizes exactly with HSP60, indicating that PC1-p15 is located within the mitochondrial matrix (Fig. 8*C*).

### PC1-p15 induces a Warburg-like metabolic phenotype

**Figure 9:**
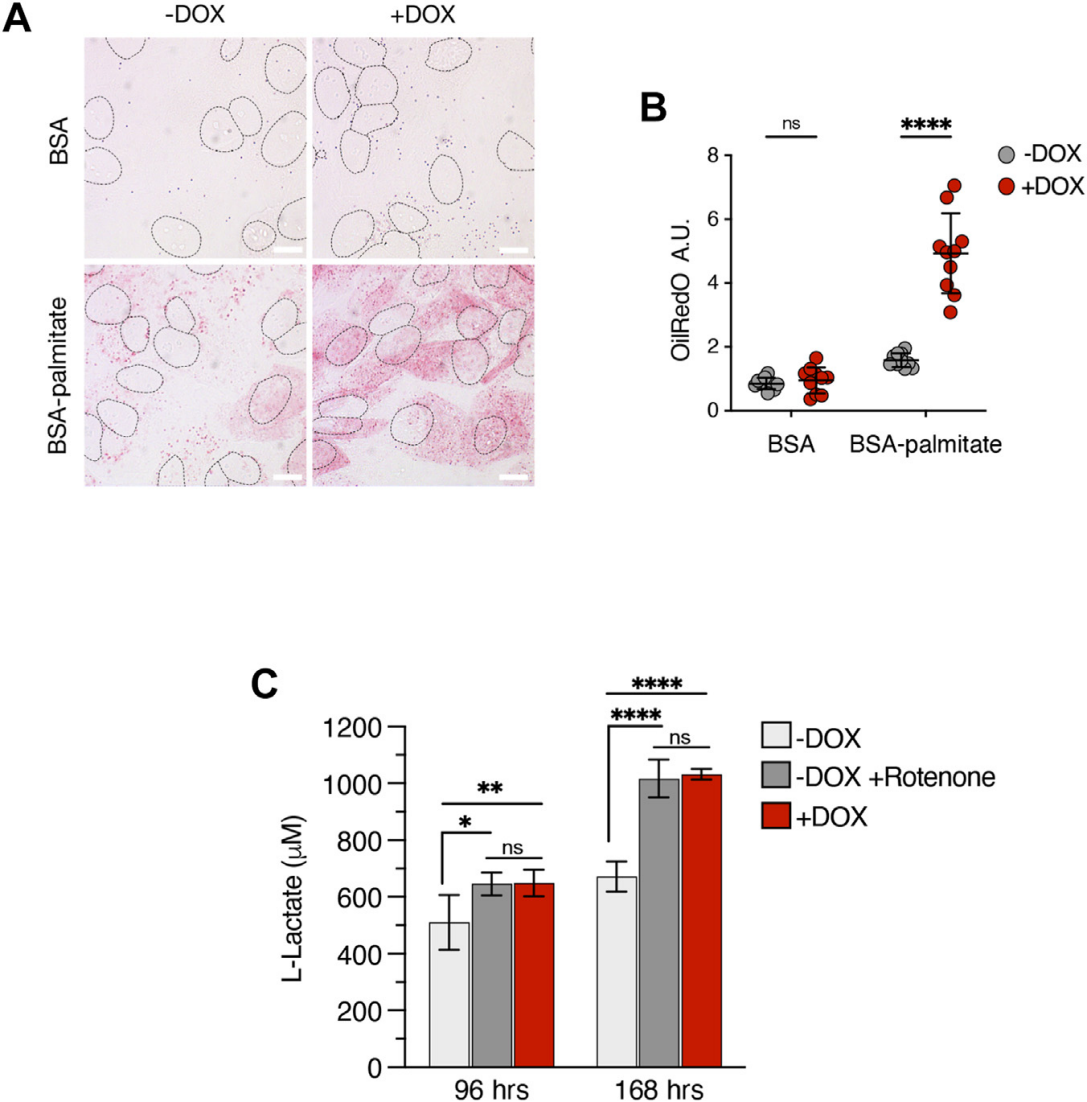
**PC1-p15 induces a Warburg-like metabolic phenotype***A*, Oil Red O neutral lipid staining of OK-p15 cells induced with DOX and treated with BSA-palmitate or BSA alone. *Outlines* represent locations of nuclei as visualized by DAPI stain. The scale bars are 10 μm. *B*, quantification of Oil Red O intensity in arbitrary units (A.U.). Data is presented as the mean ± SD, n ≥ 50 cells/condition. *C*, lactate concentration measured in cell culture medium from OK-p15 cells after treatment with DOX or the mitochondrial electron transport chain complex I inhibitor rotenone (5 μM). Cell culture media samples were collected at indicated times posttreatment, and lactate concentration was determined using a lactate standard curve. The assay was carried out with three biological and technical replicates and data is presented as the mean ± SD. (∗*p* < 0.05, ∗∗*p* < 0.01, ∗∗∗*p* < 0.001, and ∗∗∗∗*p* < 0.0001). DOX, doxycycline; OK, opossum kidney.

Since changes in mitochondrial structure are often indicative of the metabolic state of the cell, we examined fatty acid metabolism. Fatty acids are metabolized primarily by mitochondria, and an inability to break them down indicates dysfunctional mitochondria (45). PC1-p15 expression led to a dramatic increase in the number of cytosolic lipid droplets in OK-p15 cells treated with palmitic acid conjugated to bovine serum albumin (BSA-palmitate) as determined by Oil Red O staining (Fig. 9, *A* and *B*), indicative of impaired mitochondrial fatty acid oxidation. This result suggests that expression of PC1-p15 leads to the impairment of mitochondrial fatty acid oxidation, which forces the cells to convert excess fatty acids to triglycerides for storage in lipid droplets. A similar phenotype is observed in cyst-lining epithelial cells in ADPKD (46). The mitochondrial dysfunction observed in cells expressing PC1-p15 suggests that cells may adopt a Warburg-like phenotype, characterized by a metabolism reliant primarily on glycolysis for ATP production (47).

Besides mitochondrial dysfunction, a major hallmark of the Warburg effect is excessive lactate production and excretion, required to regenerate NAD+ after glycolysis (48). We used OK-p15 cells and measured extracellular lactate excretion in the cell culture media after 96 and 168 h postinduction of PC1-p15 by DOX. Cells were also treated with rotenone, a mitochondrial electron transport chain complex I inhibitor as a positive control to inhibit oxidative phosphorylation and induce a glycolytic phenotype (Fig. 9*C*). Extracellular lactate concentration significantly increased in cells expressing PC1-p15. Rotenone-induced lactate production to the same extent as PC1-p15 expressing cells, suggesting that PC1-p15 expression is sufficient to induce a glycolytic, Warburg-like phenotype that is characteristic of cyst cells in PKD.

## Discussion

**Figure 10:**
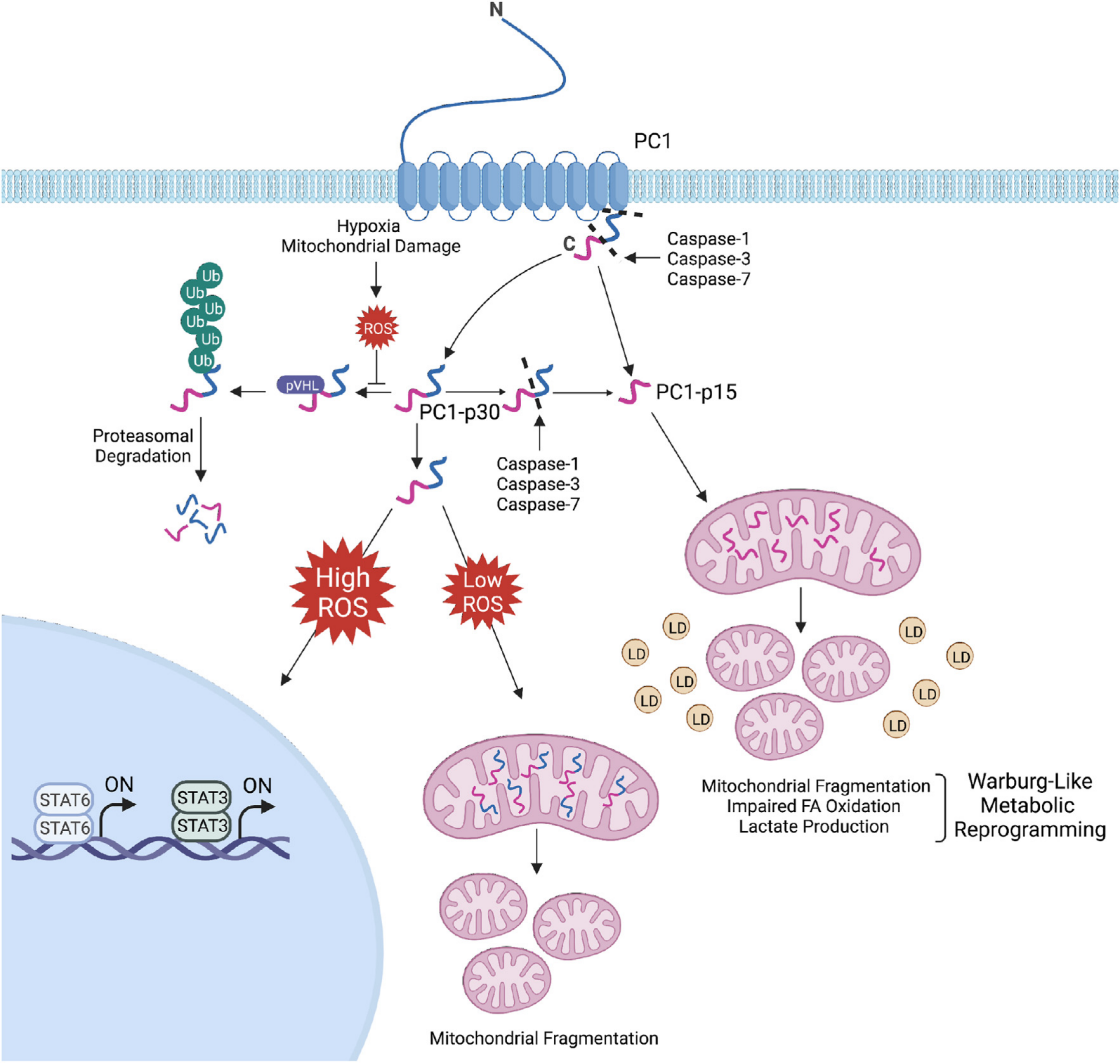
**Model of PC1 C-terminal fragments regulation and function.** Cleavage of full-length PC1 generates PC1-p30, which is rapidly degraded in the absence of ROS by the ubiquitin-proteasome system in a pVHL-dependent manner. ROS, generated in a hypoxia-dependent or hypoxia-independent manner, induce PC1-p30 stabilization. High levels of cellular ROS lead to nuclear accumulation of PC1-p30, where it can coactivate STAT3- and STAT6-dependent transcription. Low levels of cellular ROS causes PC1-p30 to target to mitochondria, where it induces mitochondrial fragmentation. Both full-length PC1 and soluble PC1-p30 can be cleaved by caspase-1, caspase-3, or caspase-7 to generate PC1-p15. PC1-p15 is constitutively localized to mitochondria, where it induces a Warburg-like metabolic phenotype as indicated by mitochondrial fragmentation, impaired fatty acid oxidation (represented by *yellow droplets*), and increased lactate production. Model created with BioRender.com. LD, lipid droplet; pVHL, von Hippel-Lindau tumor suppressor protein; ROS, reactive oxygen species; STAT, signal transducer and activator of transcription; Ub, ubiquitin.

We report herein mechanisms governing the cleavage and regulation of two distinct cleavage fragments of the PC1 C-terminal tail, and elucidate the functions of these fragments as summarized in the model (Fig. 10). PC1-p30, corresponding to the entire soluble C-terminal tail, undergoes rapid ubiquitination and proteasomal degradation. Oxidative stress (ROS) inhibits the degradation of PC1-p30, leading to its accumulation and translocation first to mitochondria and then to nuclei in a ROS concentration-dependent manner. Using a nonorthologous mouse model of PKD, we show that PC1-p30 accumulates in the nuclei of cyst-lining epithelial cells. We found that PC1-p15, which corresponds to the extreme C-terminal end of PC1, is generated by caspase-dependent cleavage of either soluble PC1-p30 or full-length membrane-bound PC1. Unlike PC1-p30, PC1-p15 is not subject to rapid degradation and constitutively localizes to mitochondria, independent of the level of oxidative stress. Overexpression of PC1-p15 leads to a Warburg-like cellular phenotype characterized by mitochondrial fragmentation, impaired fatty acid oxidation and lactate buildup. These results may help elucidate the role of the PC1 C-terminal tail in disease progression as well as the underlying cause of the mitochondrial and metabolic alterations observed in ADPKD.

Persistently elevated ROS, defined as oxidative stress, occurs in ADPKD (4, 49) and likely plays a significant role driving its disease progression (50). Oxidative stress in ADPKD occurs prior to functional impairment of the kidney and is thought to occur through multiple mechanisms (51). In line with this, we propose a mechanism to explain the presence of PC1-p30 in ADPKD: in healthy kidneys, PC1-p30 may be cleaved as part of the natural processing of PC1 but is rapidly degraded by the ubiquitin-proteasome system. In ADPKD kidneys, PC1 expression and cleavage are increased and PC1-p30 is stabilized due to the presence of ROS. The exact mechanism by which PC1-p30 cleavage is increased in ADPKD requires further investigation. A previous report provided evidence that ɣ-secretase is responsible for PC1-p30 cleavage (9), while another study found that a PC1 tail fragment accumulates in the nuclei of renal tubule cells in mice following unilateral ureteral ligation (UUO) (7). UUO, an established model of kidney injury, has been shown to induce oxidative stress, suggesting that the observed nuclear accumulation of the PC1 tail following UUO may be partially due to increased ROS (52, 53).

PC1-p30 can be further processed. We previously identified a ∼15 kDa fragment of the PC1 C-terminal tail that is abundant in kidneys from ADPKD patients, but little else was known about this fragment (8). Here, we identify a novel caspase–dependent cleavage mechanism, generating PC1-p15 from both soluble PC1-p30 and full-length membrane-bound PC1. Caspases are a family of proteases that can be divided into two groups (i) mediators of apoptosis and (ii) mediators of inflammation (54). We show that caspase-1 (an inflammatory caspase) and caspase-3 and caspase-7 (apoptotic executioner caspases) can all generate PC1-p15 by cleavage at Asp-4195 of the PC1 tail. The role of apoptosis in ADPKD remains unclear. Apoptotic caspase activity is increased in ADPKD kidneys, and caspase inhibition ameliorates renal cyst growth in some animal models (55, 56, 57). However, increased apoptosis is not a feature of PKD in mouse models, and inducing apoptosis cells can also slow disease progression (58, 59). It is therefore difficult to draw a conclusion about the role of apoptotic caspases in PC1 cleavage. Interestingly, several recent studies have demonstrated that apoptosis, once thought to be an irreversible process, can be reversed in individual cells even after activation of the executioner caspases (60, 61). Although this has yet to be shown in renal epithelial cells, apoptotic reversal has been shown to increase oncogenic capacity in several other cell types, leading to cellular changes similar to those observed in ADPKD (62, 63, 64, 65, 66). Besides apoptosis, it is well established that inflammation is a hallmark of ADPKD. Caspase-1 cleavage activates inflammatory cytokines, including IL-18, and active IL-18 is highly expressed in the kidneys, cyst fluid, and urine of ADPKD patients and animal models (67), indicating that caspase-1 is active in cyst cells. Increased caspase-1 activity may therefore account for the observed increased abundance of PC1-p15 in kidneys of ADPKD patients.

Our results indicate that these cleavage fragments of PC1 can alter mitochondrial function and cellular metabolism. Both PC1-p15 and PC1-p30 induce mitochondrial fragmentation. PC1-p15, which is constitutively targeted to the mitochondrial matrix, appears to have a more potent and robust effect and induces a Warburg-like metabolic phenotype, including impairment of fatty acid oxidation, cytosolic lipid droplet accumulation, and increased lactate production. Emerging evidence indicates that Warburg-like mitochondrial and metabolic alterations are significant factors driving disease progression in ADPKD (68, 69, 70). Impaired fatty acid oxidation is observed in animal models of PKD, suggesting that cyst cells are dependent on glucose as their main energy source as opposed to fatty acids and ketones, which require mitochondrial oxidative phosphorylation (68, 70). This metabolic inflexibility has already been successfully utilized for a therapeutic approach in PKD animal models by switching to a very low-carbohydrate diet that limits glucose availability and boosts the supply of fatty acids and ketones as alternative energy sources (70, 71). Our early clinical results suggest that ketogenic metabolic therapy may be effective in human ADPKD (72, 73) and additional clinical trials are completed, ongoing, or planned.

Of significance, mitochondrial abnormalities were shown to arise in cells carrying a heterozygous PKD1 mutation (74). The underlying cause of these impairments in PKD is unknown. Based on this evidence and the results of this study, we propose that the mitochondrial and metabolic alterations in ADPKD may be partially driven by cleavage fragments of the PC1 C-terminal tail. Early in disease progression, prior to complete loss of PKD1, the accumulation of PC1-p15 and/or PC1-p30 in cyst-lining cells may cause functional damage to the mitochondria, subsequently impairing fatty acid oxidation. This impairment forces the cells to become metabolically inflexible, primarily relying on glycolysis for ATP production and driving them toward a Warburg-like phenotype.

We and others have shown that PC1-p30 localizes to the nucleus and regulates gene transcription (6, 7, 8, 9). A previous study found that a soluble fragment of the PC1 C-terminal tail localizes to mitochondria in cell culture (29). Here, we found that PC1-p30 localizes to mitochondria under low-ROS conditions, while higher levels of ROS-induced nuclear accumulation. These findings may provide an explanation as to the different localizations reported for PC1-p30. Intrinsic cellular ROS production varies across different cell types and culture conditions, indicating that unintended ROS generation may have influenced the reported localization of PC1-p30 in previous studies. Although we cannot conclude the exact function of the differential targeting of PC1-p30, we speculate that PC1-p30 acts as a modulator of mitochondrial dynamics under low-ROS conditions and as a transcriptional modulator in high-ROS levels. It remains to be investigated which genes are regulated by this mechanism under high-ROS levels.

We show for the first time that the PC1-tail interacts with pVHL and that PC1-p30 degradation is enhanced upon binding to pVHL. Interestingly, mutations in VHL lead to renal cysts. Furthermore, like PC1, pVHL is a cilia-associated protein and was shown to regulate the formation of cilia. pVHL was also shown to physically interact with the Par3-Par6-PKC*ζ* protein complex (75). We have previously shown interaction between the PC1 tail and PKC*ζ* and a role of PKC*ζ* in cystic progression of PKD (76). It is tempting to speculate that PC1, pVHL, and PKC*ζ* may be part of a ciliary signaling complex that is involved in renal cyst growth. Further investigation seems warranted.

Although it was previously reported that PC1 is hydroxylated by PHD3 at two proline residues on the C-terminal tail (20), a modification that is necessary for recognition of HIF-1α by pVHL, PHD inhibition did not lead to PC1-p30 stabilization in our experiments. pVHL has been shown to regulate the ubiquitylation and degradation of several other proteins in a proline hydroxylation-independent manner, suggesting that PC1-p30 may be similarly regulated (77, 78, 79, 80). Alternatively, it was previously reported that the PC1 tail can be degraded through its interaction with the E3-ubiquitin ligase SIAH-1 (seven in absentia homolog-1), indicating that degradation may be mediated by multiple distinct mechanisms (81).

There are unresolved questions and incongruity in our injury model of ADPKD in which PC1-p30 and/or PC1-p15 promote cyst growth. Many pathogenic PKD1 mutations lead to truncation or loss of the PC1 cytoplasmic domain (82), meaning that PC1-p30 and PC1-p15 could not be generated from the mutated PKD1 allele. This also applies to transgenic *Pkd1*-null mouse models that clearly develop renal cysts. However, the following considerations are important to keep in mind: Cysts in ADPKD are genetically extremely diverse because only one PKD1 allele is affected by the germline mutation, while the remaining allele is either unaffected or may be mutated in a second random somatic event (83, 84, 85). Only a handful of individual cysts from ADPKD patients have been genetically investigated, and while the results were interpreted to suggest that second hit mutations are largely inactivating mutations (86), the genetic data does not necessarily agree with studies investigating PC1 expression on the protein level in which overexpression of PC1 was observed (87, 88, 89). Furthermore, transgenic overexpression of PC1 in mice causes renal cystic disease (90). Nishio *et al*. reported that cysts in chimeric Pkd1-null mice are initially formed by tubular epithelial cells with both *PKD1*^*−/−*^ and *PKD1*^*+/+*^ genotypes, and the *PKD*^*+/+*^ cells are only gradually replaced by the *PKD*^*−/−*^ cells as the cysts grow (91). It is plausible that many—and perhaps most—growing cyst cells in human ADPKD are not null for PC1 and that overexpression of PC1-p30 and/or PC1-p15 occurs as a response to oxidative stress and promote the observed Warburg-like metabolic changes that lock these cells into a glucose-dependent, metabolically inflexible state. We speculate that cleavage of the PC1 C-terminal tail is part of a discrete function of PC1, and that this mechanism is involved in furthering cyst growth and/or cyst propagation rather than initial cyst formation. In normal kidneys, the induction of PC1-p30 and/or PC1-p15 and the acquisition of the glucose-dependent metabolic state may be a beneficial, innate response of renal tubule cells to injury, involving hypoxia and oxidative stress. The glucose-dependent metabolic state would allow tubule cells to survive under hypoxic conditions and to engage in a proliferative repair program in order to recover from the injury and execute tissue regeneration. This process may be persistently activated in ADPKD, resulting in survival of cyst-lining cells in which these PC1 fragments are present and cystic progression as proposed previously (92). Further studies are required to investigate these possible mechanisms.

## Experimental procedures

### Antibodies

Anti-myc tag clone 9B11 (#ab2276; for immunofluorescence), anti-HA tag (#ab2367), and anti-HSP60 (#ab12165) were purchased from Cell Signaling Technologies. Anti-myc tag clone 9E10 (#05-419; for Western blotting), anti-β-actin (#A5441), and anti-HA (#H6908) were purchased from Millipore Sigma. Other primary antibodies included anti-HIF1-α (#10006421, Cayman Chemicals), anti-V5 tag (#R960-25, Invitrogen), anti-TOMM20 (#11802-1-AP, Proteintech), anti-Vinculin G-11 (#sc-55465, Santa Cruz Biotechnology), and anti-PC1 E8 (#E8-8C3C10, Baltimore PKD center). Rabbit antiserum against the PC1 C-terminal tail (anti-CT) was generated as described previously (8). A mouse mAb against the PC1 C-terminal tail (P175A) was generated using a recombinant human C terminally His_6_-tagged PC1-p30 antigen in collaboration with Dr B. Schermer, University Hospital Cologne. We determined that the PC1-CT antibody binds between aa 4237 to 4258 of human PC1 by antibody mapping with various deletion constructs of FLM-PC1. For immunofluorescence staining in mouse kidney sections, IgG from mouse serum (#I8765, Millipore Sigma) was used as a negative control. Secondary antibodies used for immunofluorescence: donkey-anti-mouse AlexaFluor488 (#715-545-150, Jackson ImmunoResearch), goat-anti-rabbit AlexaFluor594 (#A11012, Invitrogen), Streptavidin Dylight-594 (#21842 Invitrogen), goat-anti-mouse IgG ATTO647N (#611-156-122 Rockland Immunochemicals). Secondary antibodies used for Western blotting: goat-anti-mouse horseradish peroxidase (#115-035-044) and goat anti-rabbit horseradish peroxidase (#111-035-144) were purchased from Jackson ImmunoResearch.

### Plasmids

HA-VHL-pRc/CMV was a gift from William Kaelin (Addgene #19999), pRK5-HA-Ubiquitin-WT was a gift from Ted Dawson (Addgene #17608). Generation of FLM-PC1, PC1-P30, PC1-NTSP, PC1-CTS, and PC1-CTSP constructs was described previously (8). The PC1-p15 plasmid was made by cloning complementary DNA (cDNA) corresponding to AA 4196 to 4303 of human PC1 into a pcDNA4/TO-2myc-His backbone (Invitrogen). PC1-P15 with mitochondrial targeting sequence deletion was made by cloning cDNA sequence of aa 4220 to 4303 of human PC1 into a pCDNA4/TO-2xmyc vector. PC1-p30 cleavage mutants were made by site-directed mutagenesis of the PC1-p30 construct. For stable cell lines, cDNA of PC1-p30 or PC1-p15 was cloned into DOX-inducible pCW57-MCS1-2A-MCS2, which was a gift from Adam Karpf (Addgene #71782). Lentiviral constructs containing the sequence for human PC1-p30 or PC1-p15 under a DOX-inducible promoter were generated using the second-generation packaging plasmid pCMV-dR8.2 dvpr (Addgene #8455) and envelope plasmid pCMV-VSV-G (Addgene #8454), both gifted from Bob Weinberg. A human PC2-V5 construct was generated using a 6xMyc-PC2 plasmid provided by Gregory Germino (Johns Hopkins University) as a template and cloning it into pcDNA3.1-V5-His vector. A human PC1-FLAG plasmid and a mouse PC1 plasmid (3× FLAG on N term, 3× HA on C term) were provided by Gregory Germino and Yiqiang Cai/Stefan Somlo (Yale University), respectively. A STAT6-luciferase reporter containing five copies of a STAT6-binding element (N6-GAS) and a human STAT6 plasmid were provided by Saikh Jaharul Haque and Pankaj Sharma (Cleveland Clinic).

### Cell culture and treatments

Cell lines used included: MDCK (#CCL-34, ATCC), HEK293T (#CRL-11268, ATCC), COS-7 (#CRL-1651, ATCC), and OK (#CRL-1840, ATCC). Cells were cultured in minimum essential medium (MEM) (MDCK), Dulbecco's modified Eagle's medium (DMEM) (HEK293T, COS7), or DMEM/F-12 (OK), supplemented with 100 μg/ml Penicillin-Streptomycin (#15140122, Thermo Fisher Scientific) and 5% (MEM) or 10% (DMEM, DMEM/F-12) heat-inactivated fetal bovine serum (#FB-02, Omega Scientific). MEM was supplemented with 2 mM L-Gln (#25030081, Thermo Fisher Scientific) unless indicated otherwise. Cells were maintained at 37 °C in 5% CO_2_. DOX-inducible MDCK stable cells lines were generated as previously described (93). DOX-inducible OK stable cell lines were generated by lentiviral transfection with pCW57-MCS1-2A-MCS2-p30/p15. Transgene expression was induced using 50 ng/ml DOX (#D9891, Sigma) for 16 to 24 h prior to experimental treatments. For hypoxia induction cells were incubated for 16 h in a hypoxia chamber (Stem Cell Technologies) purged with a gas mix containing 1% O_2_, 10% CO_2_, and 89% N_2_. Chemicals and drug treatments were used at the following concentrations: CoCl_2_ (250 μM for 24 h), MG132 (1.5 μM for 24 h), CHX (50 μg/ml for 24 h), menadione (40 μM for 1 h), antimycin A (60 uM for 24 h), H_2_O_2_ (200 μM for 24 h) ALLN (25 μM for 16 h), roxadustat (100 μM for 24 h), dimethyloxalylglycine (500 μM for 24 h), L-mimosine (500 μM for 24 h), staurosporine (1 μM for 20 h), and z-VAD-fmk (20 μM for 20 h). For glutamine starvation, experiments cells were cultured in media supplemented with 2 mM L-Gln for 24 h prior to replacement with glutamine-free media.

### Western blotting

Animal tissue samples were prepared from snap-frozen whole kidneys. Tissue was mechanically homogenized and lysed in T-PER Tissue Protein Extraction Reagent (Thermo Fisher Scientific cat. #78510) (supplemented with 1 mM PMSF and 1× Halt protease inhibitor cocktail). Cultured cells were lysed in 2× Laemmli sample buffer (100 mM Tris–HCl pH 6.8, 4% SDS, 20% glycerol). Protein concentration was measured by bicinchoninic acid assay, and samples were equalized by diluting in 2× SDS sample buffer containing 0.3% bromophenol blue and 10% DTT, then boiled for 5 min at 95 °C. Proteins were separated by SDS-PAGE, transferred to a nitrocellulose (cell lysates) or polyvinylidene fluoride (tissue lysates) membrane, and incubated with blocking buffer (5% nonfat milk in 1× Tris-buffered saline, 0.1% Tween 20 detergent [TBST]). Membranes were incubated with antibodies diluted in blocking buffer and washed with 1× TBST prior to detection with an Azure 300 chemiluminescent imaging system (Azure Biosystems). For densitometric analysis, band intensities were quantified using ImageJ software (National Institutes of Health; https://imagej.nih.gov/ij/download.html) and results were normalized to β-actin loading control.

### Immunoprecipitation

Cells were transfected using Turbofect (Thermo Fisher Scientific) or treated with DOX for 18 to 24 h prior to lysis. For coimmunoprecipitation experiments, cells were lysed in lysis buffer (50 mM Tris–HCl pH 7.4, 150 mM NaCl, 1% Triton X-100, 0.1% CHAPS, 0.1% NP-40, 1 mM EDTA, 5% glycerol) or (50 mM Hepes-KOH pH 7.4, 50 mM K-acetate, 1.0% Triton X-100) containing protease and phosphatase inhibitor cocktails. For denaturing immunoprecipitation, cells were lysed in SDS lysis buffer (2% SDS, 150 mM NaCl, 10 mM Tris–HCl pH 8, 2 mM sodium orthovanadate, 50 mM sodium fluoride, protease, and phosphatase inhibitor cocktails), boiled for 10 min at 95 °C, sonicated, and diluted in dilution buffer (10 mM Tris–HCl pH 8, 150 mM NaCl, 2 mM EDTA, 1% Triton X-100). Precleared lysates were incubated with the indicated antibodies overnight at 4 °C and pulled down using protein A or G Sepharose beads. The beads were washed twice with wash buffer (Tris–HCl pH 7.4, 150 mM NaCl, 0.1% Triton X-100) and twice with wash buffer without Triton X-100. Samples were boiled in 2× Laemmli sample buffer to elute proteins from beads and subjected to SDS-PAGE and Western blot.

### Apoptotic cytosol extraction

MDCK cells were treated with 1 μM staurosporine for 4.5 h to induce apoptosis. Cells were collected in cleavage assay buffer, passed through a 27-g needle and centrifuged at 13,500 rpm for 10 min to remove cell debris. Nonapoptotic cytosol was extracted from untreated MDCK cells. The extracted cytosol was added to resin-bound PC1-p30 and the reaction was carried out in the presence of 1 mM deoxyadenosine triphosphate (Promega) for 2 h at 37 °C. Small volumes of samples were removed from the reaction at 15-min, 30-min, 1-h, and 2-h time points to examine cleavage progression.

### *In vitro* caspase cleavage assay

For *in vitro* caspase cleavage, 0.75 units of each active human recombinant caspase (BioVision, CA. cat#K233-10-25) were added to purified PC1-p30 in reaction buffer (50 mM Hepes-NaOH at pH 7.4, 50 mM NaCl, 10 mM EDTA, 5% glycerol, 10 mM DTT, and 0.1% CHAPS). The reaction was carried out for 2 h at 37 °C with moderate shaking. For a negative control, rh-caspases were pretreated with 100 μM Z-VAD-fmk (Calbiochem) for 45 min on ice. The reaction was stopped by adding SDS-containing buffer, and samples were analyzed by SDS-PAGE and Western blot.

### Luciferase assays

HEK293T cells were cultured to confluence in a 96-well plate and transfected with 40 ng human STAT6, 80 ng luciferase reporter, 3.2 ng ß-galactosidase, and either the FLM-PC1, PC1-p30 or PC1-p15 plasmid. Four h after transfection, 1 ng/ml human IL-4 (R&D Systems) and culture media without penicillin and streptomycin were added to the cells. EGFP in pCDNA4/TO was used as a negative control, and the backbone vector was used for balancing the plasmid amounts in all transfections. Luciferase assays were carried out after 20 h of treatment with IL-4 with luciferase substrate (Promega), and ß-gal activity was detected using 2-nitrophenyl β-D-galactopyranoside in sodium phosphate buffer.

### Immunocytochemistry

Cells grown on glass coverslips were fixed with 4% (w/v) paraformaldehyde for 15 min and washed with 75 mM ammonium chloride in 1× Tris-buffered saline (TBS). Cells were then incubated in blocking and permeabilization buffer (1× TBS, 0.2% Triton X-100, 2% BSA, 0.05% sodium azide) for 1 h at room temperature. Cells were incubated with primary antibodies diluted in blocking buffer overnight at 4 °C, washed three times with wash buffer (1× TBS, 0.05% Triton X-100, 0.7% fish skin gelatin), incubated with secondary antibodies diluted in blocking buffer for 1 h at 37 °C, and washed with wash buffer. Coverslips were mounted using Prolong Gold mounting reagent with 4′,6-diamidino-2-phenylindole (DAPI) (Invitrogen). For superresolution microscopy, samples were mounted in Prolong Gold mounting reagent without DAPI, and images were acquired with a stimulated emission depletion Facility microscope from Abberior Instruments.

### Oil Red O staining

OK-p15 cells were cultured in serum-free media supplemented with BSA alone or BSA conjugated to palmitate (Acros Organics cat# 416700050). After 24 h, cells were washed with 1× TBS and fixed with 4% paraformaldehyde. Cells were stained with 0.3% Oil Red O (Alfa Aesar cat#A12989) in isopropanol for 15 min and washed in TBS. Nuclei were stained with DAPI, and coverslips were mounted using glycerin jelly. At least five images/condition (>50 cells/condition) were analyzed. Images were acquired with an Olympus IX-81 microscope using a 40x objective. Oil Red O staining was quantified using ImageJ. Images were converted to grayscale images. A threshold was determined by initially applying automated thresholds to the control images (-DOX, BSA only). The cut-off values of these automated thresholds of the control were averaged, and this resulting threshold was applied to all images. Quantification data was obtained as area above threshold (arbitrary units). Area values for each image were normalized for cell count as determined by DAPI staining. A student’s *t* test was performed to test statistical significance.

### Mitochondrial morphology quantification

Images were acquired with an Olympus IX-81 fluorescence microscope with an IX2-DSU spinning disk unit (Olympus) and z-stack images were acquired using a 60×/1.4 oil objective lens. Fiji/ImageJ (https://imagej.nih.gov/ij/download.html) was used for image preprocessing. Z-projections were constructed using the SD algorithm and processed with the following methods: background subtraction, enhance local contrast (CLAHE), and white top-hat morphological filtering with the MorphoLibJ plugin (94). Mitochondrial morphology was classified using the machine learning program ilastik (95). A subset of preprocessed images were used to train the object classifier to identify three categories of mitochondrial shape (fragmented, intermediate, elongated). A minimum of 50 cells/condition were processed through the trained classifier, and images were exported with pseudocolored object classification overlays. An in-house written Matlab code was used to determine the total number of pixels in each morphological category. The percentage of mitochondria in each morphological class was calculated as the number of pixels in each class divided by the total number of pixels per image. The data is presented as the mean ± SEM.

### L-lactate assay

L-Lactate was measured in cell culture medium using a colorimetric assay as previously described (96). The lactate assay is based on the conversion of L-lactate to pyruvate by lactate dehydrogenase activity reducing NAD to NADH. NADH reduces N-methylphenazonium methyl sulphate to PMSH, which reduces p-iodonitrotetrazolium violet (INT) to INTH. Briefly, OK-p15 cells were seeded at a density of 30,000 cells/ml in complete media supplemented with 10% FBS dialyzed with a Slide-A-Lyzer Dialysis Cassette (#66810, Thermo Fisher Scientific) with a 10,000 kDA molecular weight cutoff to remove L-lactate that is present in high concentrations in serum. After 16 h, cells were induced with DOX diluted in medium or treated with 5 μM of the mitochondrial electron transport chain complex I inhibitor rotenone (#R8875, Sigma). At indicated time points postinduction, supernatant medium was taken, diluted in 1× PBS (10× dilution), and frozen at −20 °C. Thawed samples or L-lactate standards (sodium L-lactate diluted in 1× PBS) were incubated with assay buffer (86 mM triethanolamine HCl, 8.6 mM EDTA.Na_2_, 33.6 mM MgCl_2_, 326 μM N-methylphenazonium methyl sulphate, 790 μM iodonitrotetrazolium violet, 7% ethanol, 0.4% Triton X-100, 3.3 mM β-nicotinamide adenine dinucleotide, 4 U/ml L-lactate dehydrogenase) at a ratio of 1:10 sample:buffer in a 96-well plate for 30 min in the dark at room temperature. The absorbance was measured at 490 nm in a Wallac 1420 microplate reader (PerkinElmer). Absorbance values were normalized to a blank sample (cell culture media without cells), and a lactate standard curve with a spline fit curve was generated to interpolate lactate concentration in supernatant samples. Assays were carried out using at least two biological replicates and two technical replicates.

### Animal studies

All animal studies adhered to the rules and regulations of the National Institutes of Health with approval of the Institutional Animal Care and Use Committee of the University of California. The *bpk* and PKD1^flox/flox^:Tamoxifen-Cre models have been described previously (37, 97, 98). Briefly, we used a *Pkd1* inducible KO mouse model in which both alleles of PKD1 have LoxP sites flanking exons 2 to 4 under control of tamoxifen-inducible Cre recombinase (PKD1^cond/cond^). WT and *bpk*^−/−^ mice were euthanized at postnatal day 14 for analysis. For the PKD1^cond/cond^ model, Cre recombinase activity was induced by intraperitoneal injection of tamoxifen (75 mg/kg) starting at postnatal day 6, and mice were euthanized after 3 weeks. For Western blotting, snap-frozen kidney tissue was homogenized and lysed in freshly prepared tissue protein extraction reagent (Thermo Fisher Scientific). Homogenates were centrifuged and supernatants stored at −80 °C. Before loading, samples were mixed with Laemmli sample buffer and boiled at 95 °C for 5 min. For immunohistochemistry, kidneys were immediately fixed in neutral-buffered formalin and embedded in paraffin.

### Immunohistochemistry

Five micrometers thick paraffin-embedded renal tissue sections were deparaffinized and rehydrated through a standard xylene/ethanol series. Heat-induced antigen retrieval was performed for 20 min in 10 mM sodium citrate buffer (pH 6) using a pressure cooker. Endogenous biotin was blocked using the avidin/biotin blocking kit (Vector Laboratories, SP-2001). The Mouse on Mouse Immunodetection Kit (Vector Laboratories, BMK-2202) was then used according to manufacturer’s instructions. Sections were incubated with the PC1-CT mouse mAb overnight at 4 °C, washed 3 times with 1× TBST, and incubated with 1% Sudan Black B (Sigma-Aldrich #199664) to reduce autofluorescence. Following the Mouse on Mouse biotinylated anti-mouse IgG antibody, sections were incubated with Streptavidin-Dylight 594 secondary antibody for fluorescent detection. Nuclei were counterstained with DAPI, and coverslips were mounted using Prolong Diamond Antifade Mountant.

### Statistical analyses

All statistical analyses were performed using GraphPad Prism 9 software (https://www.graphpad.com/). Statistical significance was determined using unpaired, two-tailed, *t* tests with Welch’s correction for comparison between two samples, and one-way ANOVA to compare more than two samples, with *p* < 0.05 set as criteria for significance. The Tukey’s test was used to derive adjusted *p* value for pairwise comparison among multiple samples. Sample size was not predetermined.

### Study approval

All animal experiments were conducted with approval of the Institutional Animal Care and Use Committee of the University of California.

## Data availability

Full datasets used for quantifications including raw images, raw data, and code are available from the corresponding author Thomas Weimbs (weimbs@ucsb.edu) and/or Hannah Pellegrini (hannahpellegrini@ucsb.edu) upon request.

## Supporting information

This article contains supporting information.

## Conflicts of interest

T. W. is an inventor on issued and pending patents filed by the University of California, Santa Barbara related to PKD, is a shareholder of Santa Barbara Nutrients, Inc, and holds a managerial position, is a scientific advisor and shareholder of Chinook Therapeutics, received research funding from Chinook Therapeutics, and received speaker fees from Sanofi Genzyme. And also add the following sentence: All other authors declare that they have no conflicts of interest with the contents of this article.
