# Supplementary material for: Cleavage fragments of the C-terminal tail of polycystin-1 are regulated by oxidative stress and induce mitochondrial dysfunction
Source: J Biol Chem. 2023 Aug 12;299(9):105158. doi: 10.1016/j.jbc.2023.105158 (PMC10502374; doi:10.1016/j.jbc.2023.105158)
Supplement: Supplemental Figures [file mmc1.pdf]

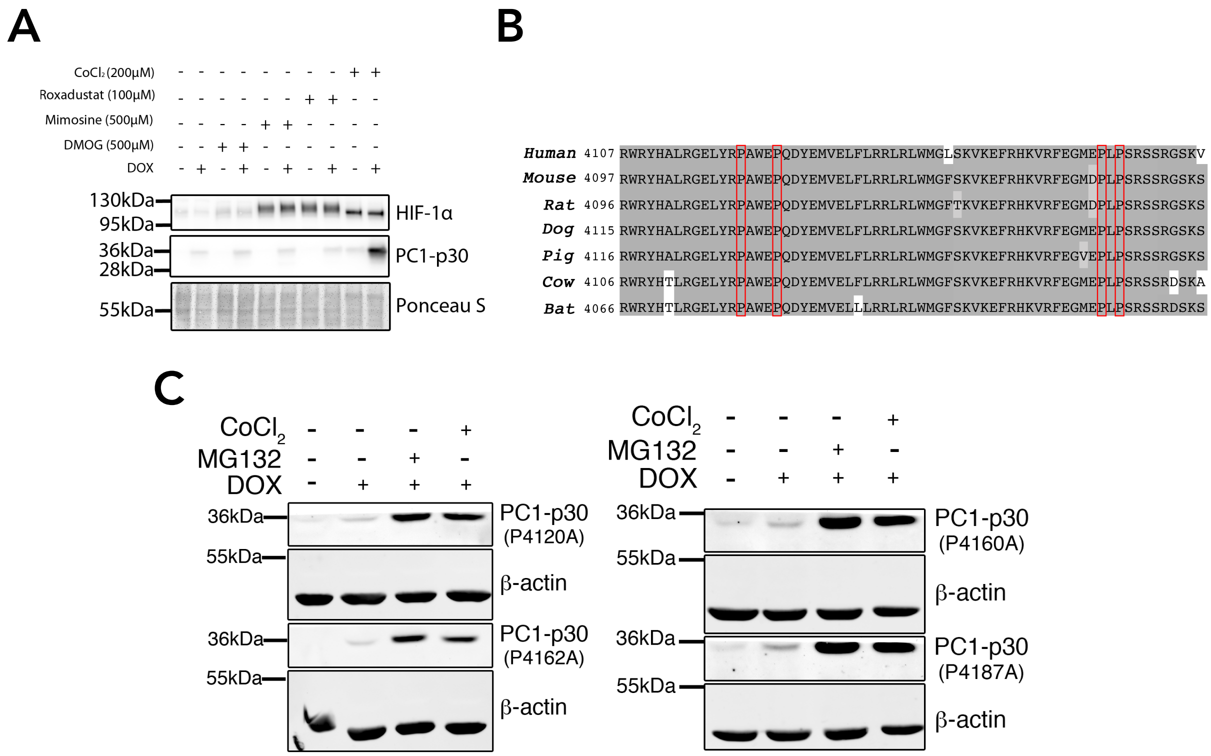

Supplemental Figure 1. PC1-p30 stabilization is independent of PHD enzyme activity and proline hydroxylation. (**A**) Immunoblot of MDCK-p30 cells treated with $CoCl_2$ or the PHD inhibitors roxadustat, mimosine, and DMOG. HIF-1α was used as a control for PHD-dependent stabilization. (**B**) Multiple sequence alignment of predicted proline hydroxylation sites in the PC1 C-terminal tail. (**C**) Immunoblot of MDCK cells transfected with PC1-p30 proline to alanine mutation constructs of the proline residues outlined in red in B. All proline mutation constructs were stabilized in the presence of $CoCl_2$ or MG132.

```
4107
RWRYHALRGELYRPAWEPQDYEMVELFLRRLRLWMGLSKVKEFRHKVRFEGMEPLPSRSS
                          ROS-M              MTS
RGSKVSPDVPPPSAGSDASHPSTSSSQLDGLSVSLGRLGTRCEPEPSRLQAVFEALLTQF
DRLNQATEDVYQLEQQLHSLQGRRSSRAPAGSSRGPSPGLRPALPSRLARASRGVDLATG
PSRTPLRAKNKVHPSST
                4303
```

Supplemental Figure 2. PC1-p30 ROS-dependent stabilization motif and mitochondrial targeting sequence. Human PC1-p30 sequence with the ROS-dependent stabilization motif (ROS-M) and mitochondrial targeting sequence (MTS) highlighted.

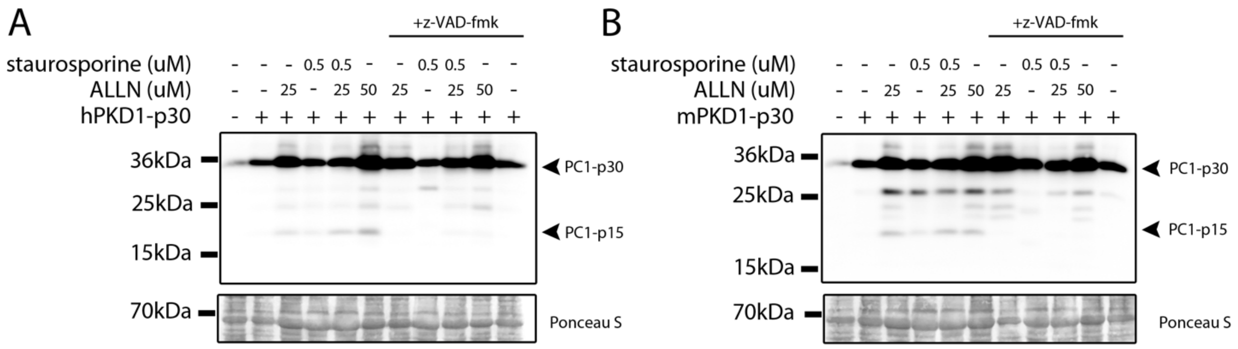

Supplemental Figure 3. PC1-p15 is cleaved from both human and mouse PC1-p30. MDCK cells were transfected with either human (**A**) or mouse (**B**) PC1-p30. PC1-p15 cleavage occurred from both human and mouse PC1-p30 in the presence of apoptosis inducers ALLN and staurosporine, and cleavage was abolished by the pan-caspase inhibitor z-VAD-fmk.
